# Infectious bursal disease virus VP5 triggers host shutoff in a transcription-dependent manner

**DOI:** 10.1128/mbio.03433-23

**Published:** 2024-01-30

**Authors:** Xinxin Niu, Jinze Han, Mengmeng Huang, Guodong Wang, Yulong Zhang, Wenying Zhang, Hangbo Yu, Mengmeng Xu, Kai Li, Li Gao, Suyan Wang, Yuntong Chen, Hongyu Cui, Yanping Zhang, Changjun Liu, Xiaomei Wang, Yulong Gao, Xiaole Qi

**Affiliations:** 1 Avian Immunosuppressive Diseases Division, State Key Laboratory for Animal Disease Control and Prevention, Harbin Veterinary Research Institute, the Chinese Academy of Agricultural Sciences, Harbin, China; 2 World Organization for Animal Health (WOAH) Reference Laboratory for Infectious Bursal Disease, Harbin Veterinary Research Institute, the Chinese Academy of Agricultural Sciences, Harbin, China; 3 Jiangsu Co-Innovation Center for the Prevention and Control of Important Animal Infectious Disease and Zoonosis, Yangzhou University, Yangzhou, China; University of Calgary, Calgary, Canada

**Keywords:** host shutoff, nucleocytoplasmic transport, Ran, RanBP1, infectious bursal disease virus

## Abstract

Viruses have evolved intricate mechanisms to evade host antiviral responses and exploit cellular resources by manipulating the expression profile of host genes. During infection, viruses encode proteins with shutoff activity to globally inhibit host protein synthesis, which is an effective strategy for immune evasion. In this study, compelling evidence shows that infectious bursal disease virus (IBDV) infection triggers the suppression of host protein synthesis. Furthermore, using both *in vitro* and *in vivo* viral infection models, we have identified that IBDV specifically impedes the transcription of host genes via the shutoff activity of viral VP5, simultaneously conferring advantages to IBDV infection in these circumstances. The proposed mechanism suggests that VP5 competitively binds to RanBP1, disrupting the RanGDP/GTP gradient. This disruption interferes with cellular nucleocytoplasmic transport, impairing the nuclear import of proteins bearing nuclear localization signals. The nuclear transport of pivotal transcriptional regulatory factors, such as p65 and IFN regulatory factor 7, is also compromised, leading to the inhibition of pro-inflammatory cytokines and interferon expression. This newly discovered strategy employed by IBDV enables them to manipulate host gene expression, providing novel insights into how viruses evade host immune responses and establish infections.

**IMPORTANCE:**

Viruses manipulate host processes at various levels to regulate or evade both innate and adaptive immune responses, promoting self-survival and efficient transmission. The “host shutoff,” a global suppression of host gene expression mediated by various viruses, is considered a critical mechanism for evading immunity. In this study, we have validated the presence of host shutoff during infectious bursal disease virus (IBDV) infection and additionally uncovered that the viral protein VP5 plays a pivotal role in inhibiting the overall synthesis of host proteins, including cytokines, through a transcription-dependent pathway. VP5 competitively binds with RanBP1, leading to disruption of the Ran protein cycle and consequently interfering with nucleocytoplasmic transport, which ultimately results in the suppression of host gene transcription. These findings unveil a novel strategy employed by IBDV to evade host innate immunity and rapidly establish infection. This study also suggests a novel supplement to understanding the pathway through which viruses inhibit host protein synthesis.

## INTRODUCTION

Viral infections trigger the host’s integrated stress response, activating antiviral immune responses upon recognizing viral nucleic acids and proteins (1). As part of this interaction, viruses manipulate host processes at various levels to regulate or evade both innate and adaptive immune responses, promoting self-survival and efficient transmission (2). In response to this host-virus arms race, viruses have evolved multiple strategies to suppress antiviral responses (3–5). One of these strategies involves the phenomenon known as “host shutoff,” a global suppression of host gene expression mediated by various viruses, which is considered a critical mechanism for evading immunity (6, 7). By inhibiting host gene expression, viruses can evade immune surveillance, block the production of antiviral factors, subvert cellular immune defenses, and redirect host resources for their replication and propagation (8).

Host shutoff encompasses diverse and intricate molecular mechanisms. Eukaryotic cells compartmentalize their nucleus within the nuclear envelope (NE), effectively separating the genome from the cytoplasmic protein synthesis machinery and allowing spatial and temporal control over transcription and translation (9). Viral proteins can directly target key host factors involved in transcription, translation, or protein modification, disrupting essential cellular protein synthesis processes. Furthermore, signal-dependent communication between the cell nucleus and cytoplasm occurs through numerous nuclear pore complexes (NPCs) spanning the NE by nucleocytoplasmic transport (NCT). Active NCT of macromolecular cargoes relies on the Ran-GTPase system, which regulates interactions between cargoes and their corresponding receptors. The conserved karyopherin-β (Kap) family of nuclear transport receptors play a central role in mediating the transportation of macromolecules, specifically proteins, in multiple orientations through the NPC, encompassing import into the nucleus (importins), export from the nucleus (exportins), and bidirectional transport (biportins) (9, 10). The Ran-GTPase assumes a pivotal role in determining the directionality of nuclear import and export mediated by Kaps. It alternates between guanosine triphosphate (GTP)-bound and guanosine diphosphate (GDP)-bound states, thereby governing cargo delivery and compartmentalization. In the cytoplasm, the GTPase-activating protein RanGAP, in cooperation with the RanGTP-binding proteins RanBP1 and RanBP2 activate GTP hydrolysis to keep cytosolic Ran in the GDP-bound state, whereas nuclear Ran is mostly bound to GTP because its guanine nucleotide exchange factor RCC1. These factors maintain a steep RanGTP-RanGDP gradient across the NE (11). To correctly transport cargo between the nucleus and cytoplasm, Kaps initially bind the cargo by recognizing linear motifs referred to as nuclear localization signals (NLSs) or nuclear export signals, as well as folded domains within their respective compartments. The Kap-cargo complex then transits the NPC through interactions with phenylalanine-glycine repeats located within the central pore’s nucleoporins. Ultimately, the cargo dissociates from the Kaps within the target compartment under the regulation of the Ran-GTPase system and recycling of the receptors for the next round of transport (9, 12, 13). This process establishes a critical bridge between host gene transcription and translation via nuclear trafficking, rendering the NPC and NCT-associated processes susceptible to viruses’ relentless efforts to curtail host protein synthesis (9, 14). The leader protein (L) encoded by encephalomyocarditis virus directly interacts with the Ran to disrupt nucleocytoplasmic transport. Consequently, host protein synthesis is inhibited, leading to adverse interferon (IFN) responses (15). In addition, viruses can silence gene expression by hijacking host signaling pathways and interfering with host epigenetic modifications (16, 17). Gaining a comprehensive understanding of how viruses regulate host gene expression is crucial for elucidating virus-host interactions, uncovering viral immune evasion mechanisms, and devising effective antiviral strategies.

Infectious bursal disease (IBD), caused by the IBD virus (IBDV), is a major immunosuppressive infectious disease that poses a serious threat to the global poultry industry (2, 18, 19). IBDV infection induces host immunosuppression by directly attacking and damaging the bursa of Fabricius, a central immune organ in chickens, resulting in increased susceptibility to other pathogenic infections and reduced vaccination efficacy (20, 21). IBDV belongs to the family *Birnaviridae*, genus *Avibirnavirus*, and is a non-enveloped, double-stranded RNA (dsRNA) virus (22–24). Its viral genome consists of two linear dsRNA segments, segment A (3.2 kb) and segment B (2.8 kb). Segment A contains two overlapping open reading frames encoding the viral structural proteins VP2 and VP3, as well as the nonstructural proteins VP4 and VP5 (23, 25–27). Segment B encodes VP1, an RNA-dependent RNA polymerase (28). Among the viral proteins encoded by IBDV, VP5 is the only non-essential protein for viral replication. Previous studies have demonstrated that the IFNs and pro-inflammatory mediators, such as cytokines, chemokines, and antimicrobial peptides, effectively limits and clears IBDV viral infection (2, 29). Recently, single-cell sequencing studies have shown that intranasal inoculation with IBDV allows the virus to quickly traverse the immune barrier, accessing the target organ, bursa, and establishing infection (30). However, the detailed mechanisms by which IBDV breaks through the host’s line of defense, evade immunity, and rapidly establishes infection in competition with host immune responses remain unclear.

In this study, we have validated the presence of host shutoff during IBDV infection and additionally uncovered that the viral protein VP5 plays a pivotal role in inhibiting the overall synthesis of host proteins, including cytokines, through a transcription-dependent pathway. VP5 competitively binds with RanBP1, leading to disruption of the Ran protein cycle and consequently interfering with nucleocytoplasmic transport, which ultimately results in the suppression of host gene transcription. These findings unveil a novel strategy employed by IBDV to evade host innate immunity and rapidly establish infection. This study also suggests a novel supplement to the pathway through which viruses inhibit host protein synthesis.

## RESULTS

### VP5 of IBDV induces a global shutoff of host protein synthesis

**Fig 1:**
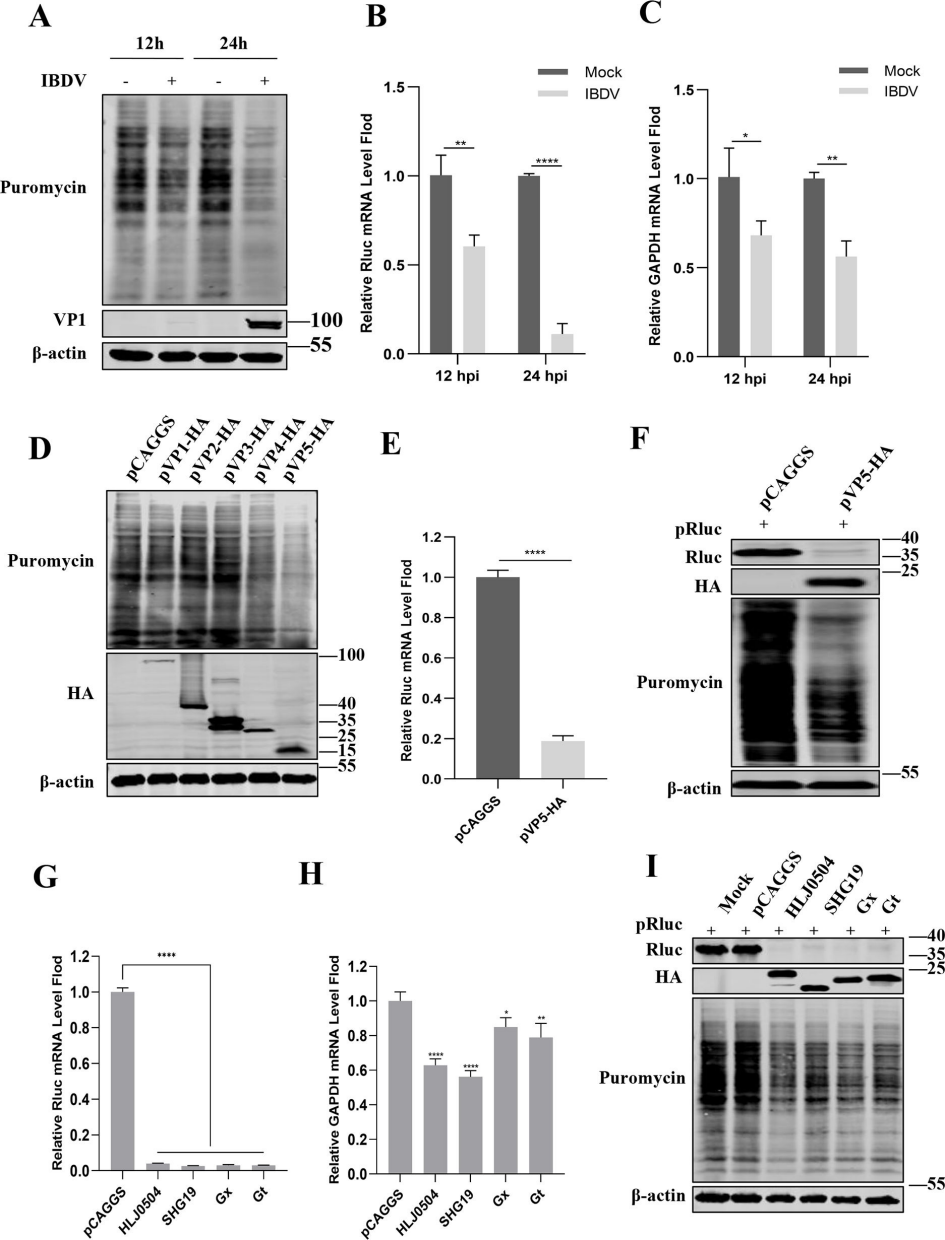
IBDV VP5 is a critical viral agent triggering the shutoff of host protein synthesis. (**A**) The ribopuromycylation assay was conducted in DF1 cells infected with IBDV. When DF1 cells were cultured on a culture plate for 12 h, cells were infected with IBDV at an MOI of 1 for 12 h and 24 h, and then puromycin was added to the cells for 25 min prior to whole-cell lysate collection. Western blotting was used to detect IBDV VP1, β-actin, and puromycin for evaluating nascent host proteins. A non-infected DF1 cell control was also included. (**B and C**) The impact of IBDV on exogenous Rluc (**B**) or endogenous GAPDH (**C**) synthesis was assessed. DF1 cells were transfected with the recombinant plasmid expressing Rluc (pRluc) for 12 h and then infected with IBDV at an MOI of 1. At 12 and 24 hpi, total cell RNA was extracted, and Rluc and GAPDH mRNA levels were measured by RT-qPCR. (**D**) The ribopuromycylation assay was performed in DF1 cells transfected with viral proteins of IBDV. Recombinant plasmids expressing viral proteins (VP1–VP5) or an empty vector (pCAGGS) were transfected into DF1 cells. At 36 hpi, puromycin was added for 25 min, and Western blotting was conducted using an anti-puromycin antibody. (**E and F**) The influence of IBDV VP5 on host protein synthesis was investigated. Recombinant plasmids expressing viral VP5 [pVP5-hemagglutinin (HA)] and Rluc were transfected into DF1 cells. The transfection dose of each plasmid is 1 µg /5 × 10^5^ cells. At 36 hpi, Rluc mRNA was measured using RT-qPCR (**E**), and the expressions of Rluc and puromycin were measured using Western blotting (**F**). (**G–I**) The shutoff activity of VP5 from different representative strains of IBDV in DF1 cells was also assessed by detecting exogenous Rluc mRNA (**G**), endogenous GAPDH mRNA (**H**), and host protein synthesis (**I**). *P < 0.05, **P < 0.01, ***P < 0.001, and ****P < 0.0001.

The inhibition of host protein synthesis serves as a successful viral evolutionary strategy to counteract host antiviral responses and establish infection (31). In this study, we observed a broad-spectrum inhibitory activity of IBDV infection on host protein synthesis. When chicken fibroblast cells (DF1) were infected with IBDV at a multiplicity of infection (MOI) of 1 for 12 h or 24 h, the newly synthesized proteins were examined using a ribopuromycylation assay to monitor the global efficiency of cellular protein synthesis. The data showed that the broad-spectrum inhibitory activity of IBDV on host protein synthesis was dependent on the duration of infection and viral accumulation (Fig. 1A). To further explore the mechanism by which viral infection triggers shutoff, we assessed the mRNA levels of the exogenous reporter gene encoding Renilla luciferase (Rluc) and the host endogenous gene encoding glyceraldehyde-3-phosphate dehydrogenase (GAPDH) during IBDV infection using reverse transcription quantitative real-time PCR (RT-qPCR). At 12 h and 24 h post-infection (hpi), the mRNA levels of Rluc (Fig. 1B) and GAPDH (Fig. 1C) were significantly reduced in the IBDV-infected cells compared to the mock-infected samples.

To assess the effect of viral proteins on host protein synthesis, we verified the IBDV viral proteins separately by ribopuromycylation assay. The results revealed that the IBDV nonstructural protein VP5 played a key role in suppressing host protein synthesis (Fig. 1D). Subsequent RT-qPCR experiments showed that the overexpression of VP5 significantly reduced the mRNA levels (Fig. 1E) and protein abundance (Fig. 1F) of the Rluc reporter. To investigate whether this phenomenon is common across IBDV strains, we transfected DF1 cells with recombinant plasmids expressing representative VP5 from various IBDV laboratory strains and clinical isolates. The results demonstrated that VP5 from different IBDV strains decreased the mRNA levels of the exogenous Rluc reporter (Fig. 1G) and endogenous GAPDH (Fig. 1H), which was also evident at the protein synthesis and accumulation levels (Fig. 1I). In conclusion, IBDV triggers the shutoff of host protein synthesis, and VP5 is identified as a critical factor.

### VP5 with a shutoff function is beneficial to viral replication

**Fig 2:**
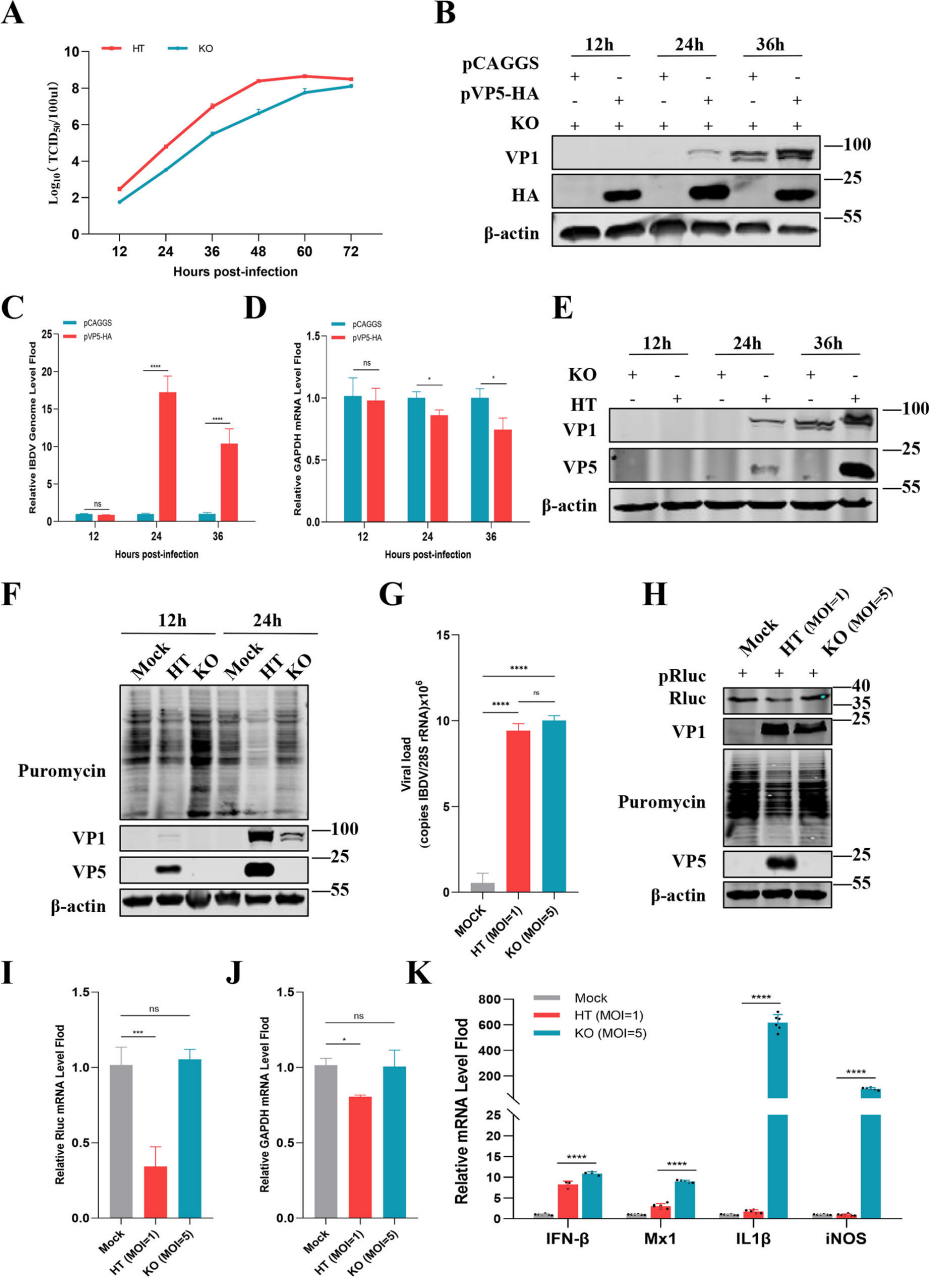
VP5 with shutoff function enhances virus replication. (**A**) Replication kinetics curves of the HT strain and KO strain in DF1 cells. Cells were infected with the viruses at an MOI of 0.01 for 12 h, 24 h, 36 h, 48 h, 60 h, and 72 h. The virus titers of 50% tissue infection dose (TCID_50_) were titrated in DF1 cells. (**B–D**) Anaplerotic experiment of VP5. DF1 cells were transfected with pVP5-HA or the empty vector for 12 h and then infected with the KO strain at an MOI of 0.1. At 12, 24, and 36 hpi, viral proteins were measured by Western blotting (**B**), while viral RNA (**C**) and GAPDH mRNA (**D**) were detected by RT-qPCR. (**E**) DF1 cells were infected with the KO or HT strain at an MOI of 0.1. At 12, 24, and 36 hpi, viral proteins were measured by Western blotting. (**F–K**) Comparison of shutoff activity between IBDV strains with or without VP5. Ribopuromycylation assay in DF1 cells infected with the HT strain and KO strain. DF1 cells were infected with HT or KO strains at an MOI of 1 for 12 h and 24 h. Puromycin was added to the cells for 25 min prior to whole-cell lysate collection, and VP1, β-actin, and puromycin were detected using Western blotting (**F**). Comparison of shutoff activity between the HT strain and KO strain at 24 hpi with the same viral titer (**G–K**). DF1 cells were transfected with recombinant plasmid expressing Rluc (pRluc) for 12 h and then infected with the HT strain at an MOI of 1 or the KO strain at an MOI of 5. At 24 hpi, viral genome copies were measured by RT-qPCR (**G**). Puromycin was added to the cells for 25 min, and Rluc, VP1, β-actin, and puromycin were measured using Western blotting (**H**). In the experiment of Fig. 2G, the mRNA levels of exogenous Rluc (**I**), endogenous GAPDH (**J**), and cytokines [IFN-β, Mx dynamin-like GTPase 1 (Mx1), inducible nitric oxide synthase (iNOS), and interleukin-1β (IL-1β)] (**K**) in cells at 24 hpi were detected by RT-qPCR. *P < 0.05, **P < 0.01, ***P < 0.001, ****P < 0.0001; ns, not significant.

Viruses are obligate intracellular parasites, and their lifecycle ultimately depends on host cells. To investigate the influence of VP5 with the shutoff activity on viral replication, we generated the VP5-deletion strain (KO) based on its parental IBDV strain (HT) and confirmed their molecular characteristics through sequencing. Initially, we compared the replication kinetics of HT and KO strains. The results revealed a notable delay in the replication of the KO strain compared to the HT strain in DF1 cells, with the titer of KO strain being approximately 57.5-fold lower than that of HT strain at 48 hpi, underscoring the critical role of VP5 in influencing IBDV replication efficiency (Fig. 2A). To further validate the role of VP5 in host shutoff and IBDV replication, we conducted an anaplerotic experiment with VP5. DF1 cells were transfected with pVP5-hemagglutinin (HA) or an empty vector (pCAGGS) for 12 h, infected subsequently with IBDV KO strain, and then viral protein and RNA were detected by Western blotting and RT-qPCR at 12, 24, and 36 hpi. The data demonstrated a significant increase in viral protein at 24 and 36 hpi in the pVP5-HA transfected group compared to the empty vector group (Fig. 2B). Similarly, the viral RNA levels were significantly elevated in the pVP5-HA transfected group at 24 and 36 hpi (Fig. 2C). Furthermore, the detection of endogenous GAPDH in the same experiment confirmed the inhibitory effect of VP5 on host gene expression (Fig. 2D). Next, DF1 cells were infected with HT or KO strains, and a comparative analysis of viral protein expression was carried out using Western blotting at 12, 24, and 36 hpi. Specifically, the group infected with the HT strain exhibited significantly higher viral protein levels compared to the KO strain. This difference was particularly pronounced at 24 and 36 hpi (Fig. 2E). Overall, these data indicate that VP5 is beneficial for viral protein synthesis and virus replication while it concurrently inhibits host protein synthesis.

In subsequent investigation, we aimed to elucidate the mechanism by which VP5 triggers host shutoff. This endeavor seeks to enhance our comprehension of why VP5 promotes viral replication while concurrently impeding host protein synthesis. The effects of VP5 on host protein synthesis at the virus level were compared systematically. DF1 cells were infected with HT or KO strains at an MOI of 1 for 12 h and 24 h, and the newly synthesized proteins were then examined using a ribopuromycylation assay. The Western blotting analysis revealed that HT strain infection significantly reduced host protein synthesis, whereas KO strain infection had no significant effect compared to uninfected cells (Fig. 2F). To minimize detection errors induced by differences in replication efficiency and the final titer of the two strains, we conducted another infection experiment using a fivefold higher infection dose of KO than HT strain, ensuring that both strains had similar titers at 24 hpi, as shown in Fig. 2G. The abundance of protein and mRNA in infected cells was examined. The results demonstrated that VP5 deletion in the KO strain restored the protein abundance of Rluc (Fig. 2H), the mRNA levels of Rluc (Fig. 2I), and GAPDH (Fig. 2J), which had been suppressed by HT strain infection.

Innate immune responses, including IFNs signaling and inflammatory responses, play essential roles in combating IBDV infection (2). To investigate the impact of impaired host protein synthesis on the innate immune responses to IBDV infection, we also examined the transcriptional activation of IFN-β, Mx dynamin-like GTPase 1 (Mx1), inducible nitric oxide synthase (iNOS), and interleukin-1β (IL-1β) induced by HT or KO strains at the same final titer in DF1 cells (Fig. 2G). The results demonstrated that HT strains triggered lower levels of IFN-β, Mx1, iNOS, and IL-1β compared to KO strains (Fig. 2K). Clearly, VP5-induced host shutoff leads to the suppression of major cytokine synthesis in IBDV-infected cells, demonstrating an important mechanism for IBDV to inhibit host immune responses and enhance viral infection.

### VP5-induced repression of host protein synthesis is independent of cellular protein degradation and protein translation

**Fig 3:**
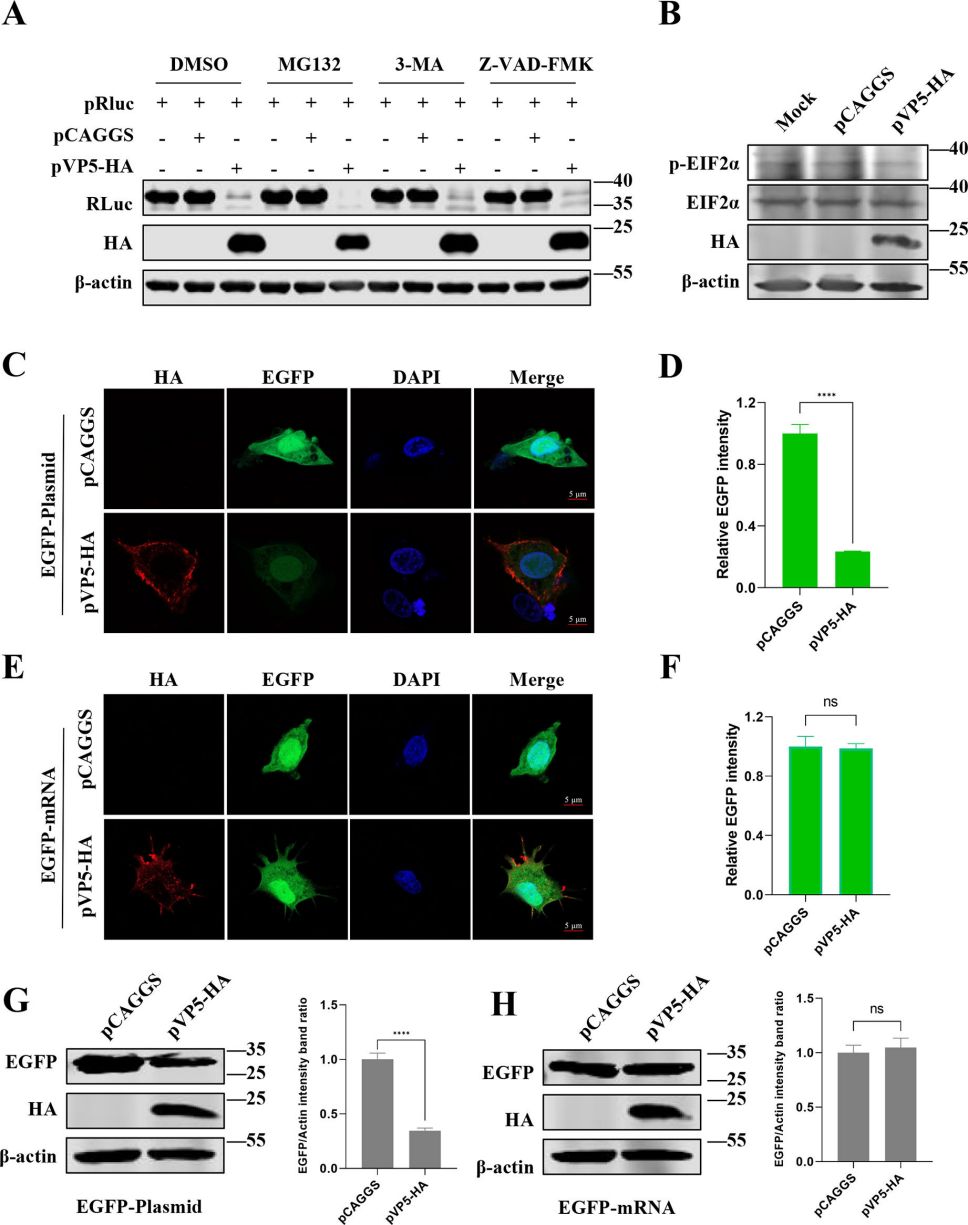
The shutoff activity of viral VP5 is independent of intracellular protein degradation systems and protein translation. (**A**) DF1 cells were co-transfected with recombinant plasmids expressing Rluc and viral VP5 or an empty vector for 24 h, and then the cells were treated with DMSO, proteasome inhibitor (MG132), autophagy inhibitor (3-MA), or apoptosis inhibitor (Z-VAD-FMK) for 12 h. Protein expression was detected using Western blotting. (**B**) DF1 cells were transfected with pVP5-HA, pCAGGS, or mock for 24 h, and then cell lysates were harvested and detected by Western blotting using anti-HA, anti-p-eukaryotic initiation factor 2α (eIF2α), anti-eIF2α, and anti-β-actin antibodies. (**C**) Representative images of DF1 cells co-transfected with recombinant plasmids expressing enhanced green fluorescent protein (pEGFP-C1) and either pVP5-HA or pCAGGS. The VP5 was detected by indirect immunofluorescence (red), and EGFP was shown by live fluorescence (green). Cell nuclei were counterstained with DAPI (4′,6-diamidino-2-phenylindole), (blue). (**D**) Quantification of relative EGFP intensity corresponding to Fig. 3C. (**E**) Representative images of DF1 cells co-transfected with EGFP mRNA and either pVP5-HA or pCAGGS. (**F**) Quantification of relative EGFP intensity corresponding to Fig. 3E. (**G and H**) The cell samples collected in Fig. 3C (**G**) and Fig. 3E (**H**) were also used to quantify indicated protein expression using Western blotting. ****P < 0.0001; ns, not significant.

To investigate whether the host shutoff induced by viral VP5 is linked to intracellular protein degradation pathways, DF1 cells were transfected with either pVP5-HA or an empty vector, and then treated with dimethyl sulfoxide (DMSO), proteasome inhibitor (MG132), autophagy inhibitor (3-MA), or apoptosis inhibitor (Z-VAD-FMK), respectively. The results demonstrated that the three inhibitors could not effectively restore the inhibition of VP5 on the abundance of the Rluc reporter, indicating that VP5-induced repression of host protein accumulation was independent of cellular protein degradation (Fig. 3A).

To verify the effect of VP5 on protein translation, we investigated whether VP5 enhances eukaryotic initiation factor 2α (eIF2α) phosphorylation (Fig. 3B). The results revealed that the overexpression of VP5 did not induce an increase in phosphorylated eIF2α (p-eIF2α) compared to the empty vector group and the untransfected group. Furthermore, DF1 cells were co-transfected with an EGFP reporter plasmid (pEGFP-C1) and either pVP5-HA or pCAGGS, and then imaged EGFP fluorescence and anti-HA immunofluorescence. The confocal laser scanning results showed that EGFP expression in cells expressing VP5 was reduced by over 5.8-fold (*P* < 0.001) compared to the empty vector group (Fig. 3C and D). Meanwhile, when the mRNA of EGFP replaced the EGFP reporter plasmid, no significant difference in EGFP expression was observed after co-expression with VP5 or the empty vector (Fig. 3E and F), indicating that the shutoff activity of VP5 is independent of translation. The corresponding Western blotting experiments (Fig. 3G and H) were consistent with the above results. Thus, these data indicated that the shutoff activity of viral VP5 is independent of intracellular protein degradation pathways or protein translation stage.

### VP5 induces the shutoff of host protein synthesis in a transcription-dependent manner

**Fig 4:**
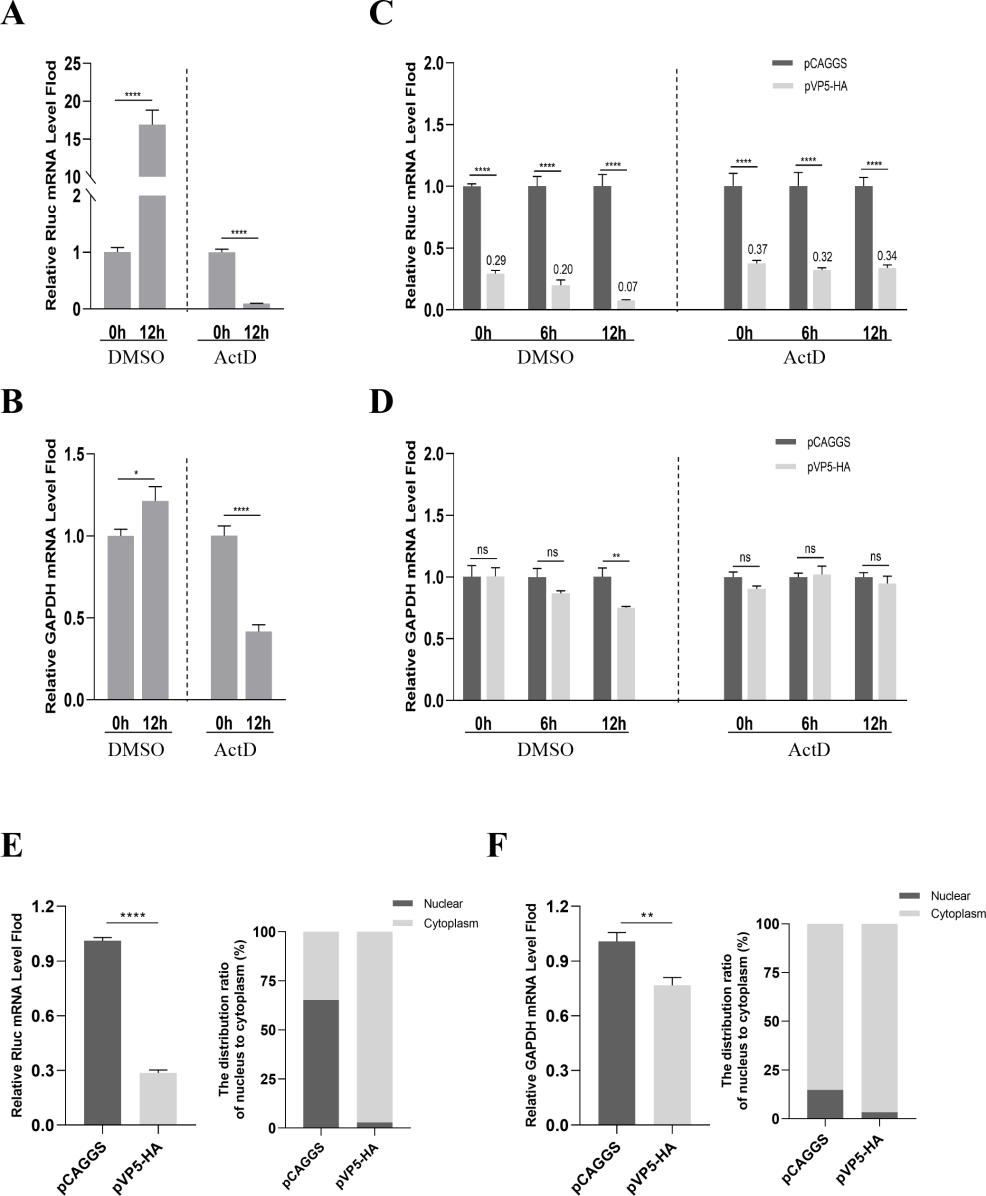
VP5 represses host genes transcription. (**A and B**) DF1 cells were transfected with pRluc for 24 h (0 h) and then treated with ActD (2 µg/mL) or DMSO for another 12 h. Cellular RNA was extracted, and the levels of Rluc mRNA (**A**) and GAPDH mRNA (**B**) were determined by RT-qPCR. (**C and D**) DF1 cells were co-transfected with pRluc and either pVP5-HA or pCAGGS for 24 h and then treated with ActD (2 µg/mL) or DMSO for another 12 h. Cellular RNA was extracted at 0 h, 6 h, and 12 h after ActD treatment, and the levels of Rluc mRNA (**C**) and GAPDH mRNA (**D**) were examined by RT-qPCR. The DMSO-treated group was included as a control. (**E and F**) Furthermore, DF1 cells were co-transfected with pRluc and either pVP5-HA or pCAGGS for 36 h. Cytoplasmic and nuclear RNA were extracted separately, and the levels of Rluc and GAPDH mRNA were determined by RT-qPCR. Then, the ratios of Rluc and GAPDH mRNA in nuclear were statistically analyzed. *P < 0.05, **P < 0.01, ***P < 0.001, ****P < 0.0001; ns, not significant.

Macroscopically, protein synthesis involves two stages: transcription and translation. To investigate the effect of VP5 on transcription and mRNA stability, we first verified that treatment with actinomycin D (ActD) blocked the mRNA synthesis of exogenous Rluc (Fig. 4A) and endogenous GAPDH (Fig. 4B) in DF1 cells. In another experiment, DF1 cells were co-transfected with recombinant plasmid expressing Rluc (pRluc) and pVP5-HA or pCAGGS for 24 h and then treated with ActD or DMSO. Total RNA was collected at 6-h intervals and analyzed by RT-qPCR. The analysis of the results showed a significant decrease in Rluc mRNA levels in the VP5-expressing cells compared to the empty vector group within the DMSO-treated control group.

Moreover, this reduction progressively increased with time (Fig. 4C). However, when treated with ActD to repress host transcription, the accumulation of the reduction in Rluc mRNA over time ceased to occur (Fig. 4C). Additionally, similar to the Rluc reporter, the time-concomitant decrease of endogenous GAPDH mRNA in the VP5-expressing group was not observed with ActD treatment (Fig. 4D). Therefore, VP5 inhibits host mRNA accumulation by affecting transcription rather than promoting mRNA degradation. As mRNA transcription primarily occurs in the nucleus, we further examined the effect of VP5 on mRNA of Rluc and GAPDH in the nucleus. DF1 cells were co-transfected with pRluc and either pVP5-HA or pCAGGS for 36 h. Afterward, whole-cell RNA was extracted and measured, and the results showed that VP5 significantly suppressed the abundance of both Rluc (Fig. 4E left) and GAPDH mRNA levels (Fig. 4E left). In addition, another set of samples with the same experimental conditions was processed to extract cytoplasmic and nuclear RNA separately and then assessed through RT-qPCR. Subsequently, we calculated and compared the changes in the distribution ratio of mRNA for Rluc and GAPDH in the nucleus of the VP5 expression group compared to the control group. The results revealed that VP5 significantly suppressed the proportion of nuclear mRNA of both Rluc (Fig. 4E right) and GAPDH (Fig. 4F right). These data further indicate that VP5 induces host protein synthesis shutoff by inhibiting host gene transcription.

### VP5 disrupts host transcriptional regulation by interfering with nucleocytoplasmic transport

**Fig 5:**
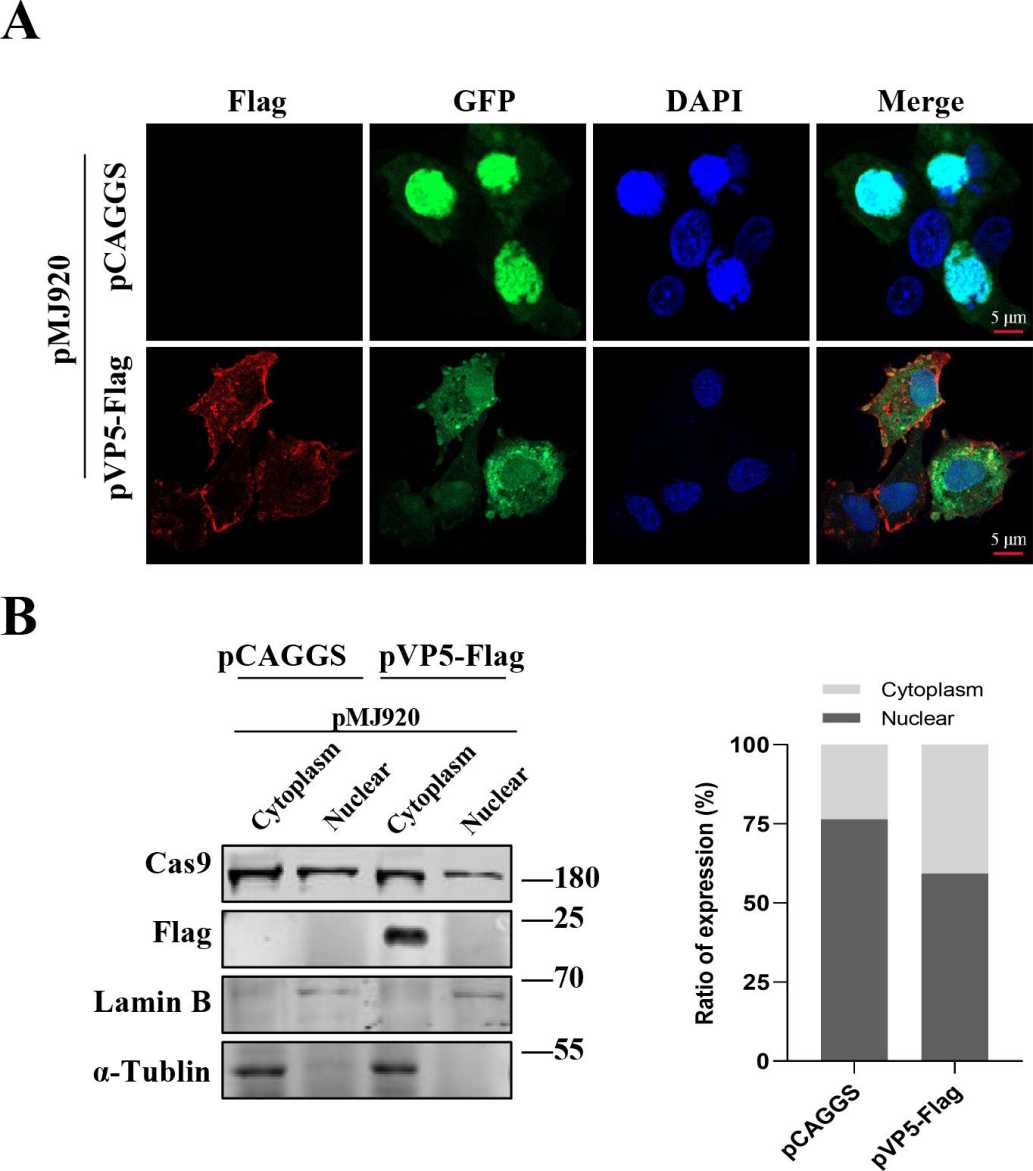
VP5 disrupts host nucleocytoplasmic transport. (**A**) DF1 cells were co-transfected with pMJ920 and either pVP5-Flag or pCAGGS for 24 h. The cells were fixed and subjected to labeling with mouse anti-Flag antibodies to visualize VP5 proteins (red) by immunostaining and EGFP-Cas9 by *in situ* fluorescence (green). (**B**) Another set of samples under the same experimental conditions as Fig. 5A was separated into cytoplasmic and nuclear extracts. The protein abundance of Cas9 in cytoplasmic and nuclear extracts was analyzed via Western blotting.

Although VP5 can influence the nuclear transcription events, it does not enter the nucleus during viral infection. Transcription depends on the active participation of the cellular cytoplasmic transport system, completing material exchange and signal transmission. Therefore, we speculate that viral VP5 may manipulate transcription in the nucleus by interfering with the nucleocytoplasmic transport through certain mechanisms. To validate the hypothesis, DF1 cells were co-transfected with pMJ920, the plasmid fusion expressing green fluorescent protein (GFP) and Cas9 with SV40-NLS, and either pVP5-Flag or pCAGGS for 24 h. Afterward, the effect of VP5 on Cas9 entry into the nucleus was observed by confocal microscopy experiments (Fig. 5A). The results indicated that VP5 can significantly inhibit the nuclear transport of Cas9 protein. In subsequent experiments on protein nucleocytoplasmic separation, the results also confirmed this conclusion (Fig. 5B). In summary, we demonstrate that VP5 is able to significantly impede nucleocytoplasmic transport, which may be a critical mechanism by which VP5 inhibits host transcription.

**Fig 6:**
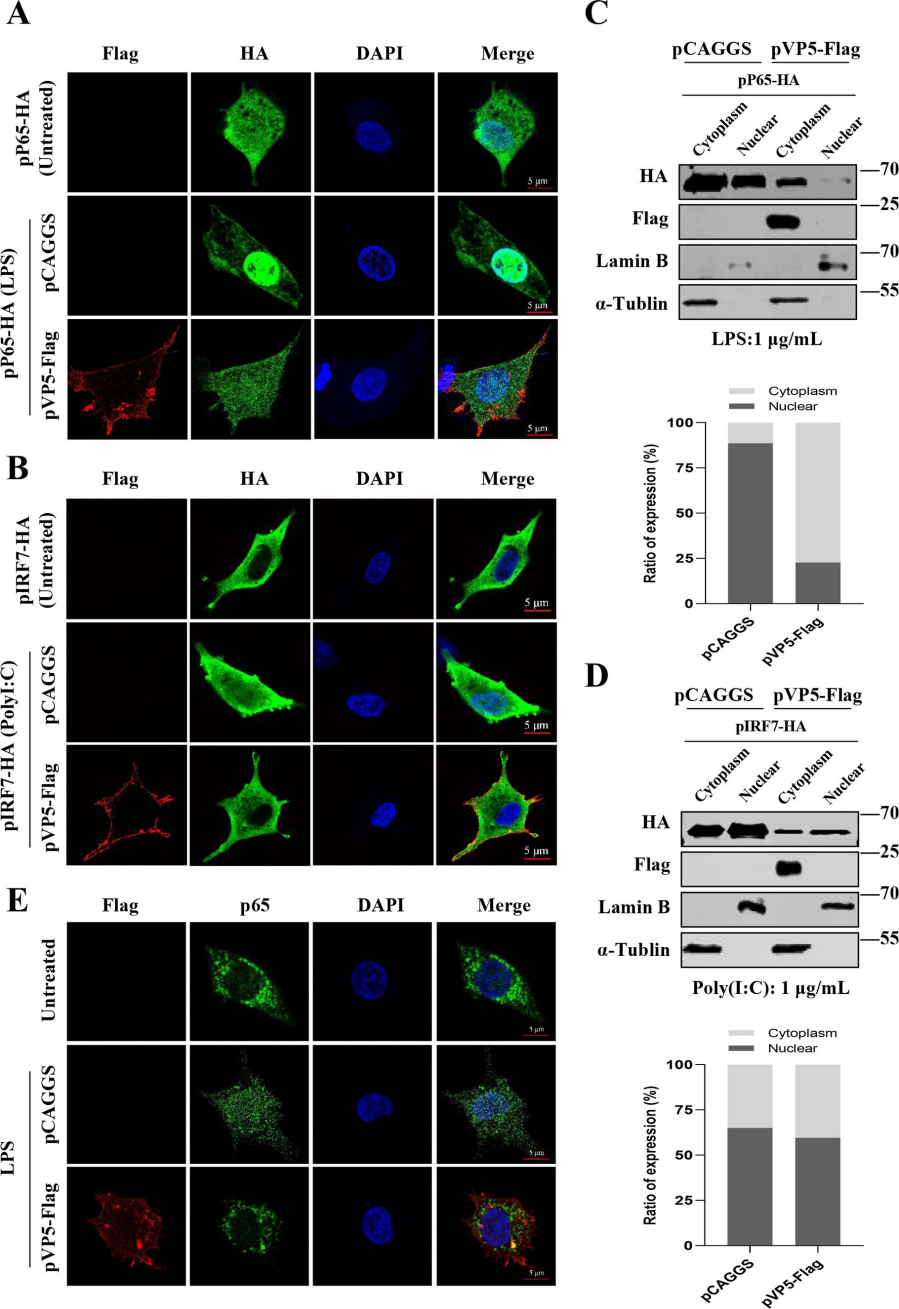
VP5 manipulates host transcriptional regulation by interfering with nucleocytoplasmic transport. (**A**) DF1 cells were transfected with an empty vector or pVP5-Flag for 8 h, followed by transfection with a recombinant plasmid expressing p65 (pP65-HA) for 24 h, and then treated with LPS (1 µg/mL) or mock for 2 h. The cells were fixed and subjected to dual labeling with mouse anti-HA and rabbit anti-Flag antibodies to visualize p65 (green) and VP5 (red) by immunostaining. (**B**) DF1 cells were transfected with an empty vector or pVP5-Flag for 8 h, followed by transfection with a recombinant plasmid expressing IRF7 (pIRF7-HA) for 24 h, and then treated with transfection of poly(I:C) (1 µg/mL) or mock for 12 h. The cells were fixed and subjected to dual labeling with mouse anti-HA and rabbit anti-Flag antibodies to visualize IRF7 (green) and VP5 (red) by immunostaining. (**C and D**) Another set of samples under the same experimental conditions as Fig. 6A or 6B was separated into cytoplasmic and nuclear extracts. The protein levels of p65 (**C**) or IRF7 (**D**) in cytoplasmic and nuclear extracts were analyzed via Western blotting. (**E**) DF1 cells were transfected with an empty vector or pVP5-Flag for 24 h and then treated with LPS (1 µg/mL) for 2 h and set up a control group. The cells were fixed and subjected to dual labeling with rabbit anti-p65 and mouse anti-Flag antibodies to visualize p65 (green) and VP5 (red) by immunostaining.

In this study, we demonstrated that VP5, with a shutoff function, significantly restricts the production of IFN and IFN-stimulated genes (ISGs) caused by viral infection, along with the expression of related inflammatory factors (IL-1β, iNOS) (Fig. 2K). To further clarify the effect of VP5 on nucleocytoplasmic transport and host transcription, confocal microscopy experiments were performed to investigate whether VP5 affects the nucleocytoplasmic distribution of the transcription factors nuclear factor (NF)-κB p65 subunit and IFN regulatory factor 7 (IRF7, which regulate the transcription of inflammation-related and IFN genes. The results revealed that VP5 affected the nucleocytoplasmic distribution of p65 (Fig. 6A; Fig. S1A) and IRF7 (Fig. 6B; Fig. S1B) and demonstrated a more pronounced inhibition of p65 nuclear import. Furthermore, the proteins were separately extracted from the cytoplasm and the nucleus, and the corresponding Western blotting results validated the above findings. Consistently, VP5 inhibited the nuclear translocation of p65 (Fig. 6C) and IRF7 (Fig. 6D) following stimulation with lipopolysaccharide (LPS) or polyinosinic:polycytidylic acid [poly(I:C)]. Next, we examined the influence on the nucleocytoplasmic distribution of endogenous p65, as illustrated in Fig. 6E; Fig. S1C. Notably, while LPS stimulation promoted p65 entry into the nucleus, VP5 exhibited a remarkable capacity to inhibit this translocation significantly. These results suggest that the transcription-dependent shutoff activity of VP5 may be achieved by interfering with nucleocytoplasmic transport.

### VP5 disrupts Ran cycling and interferes with nucleocytoplasmic transport by competitively binding to RanBP1

**Fig 7:**
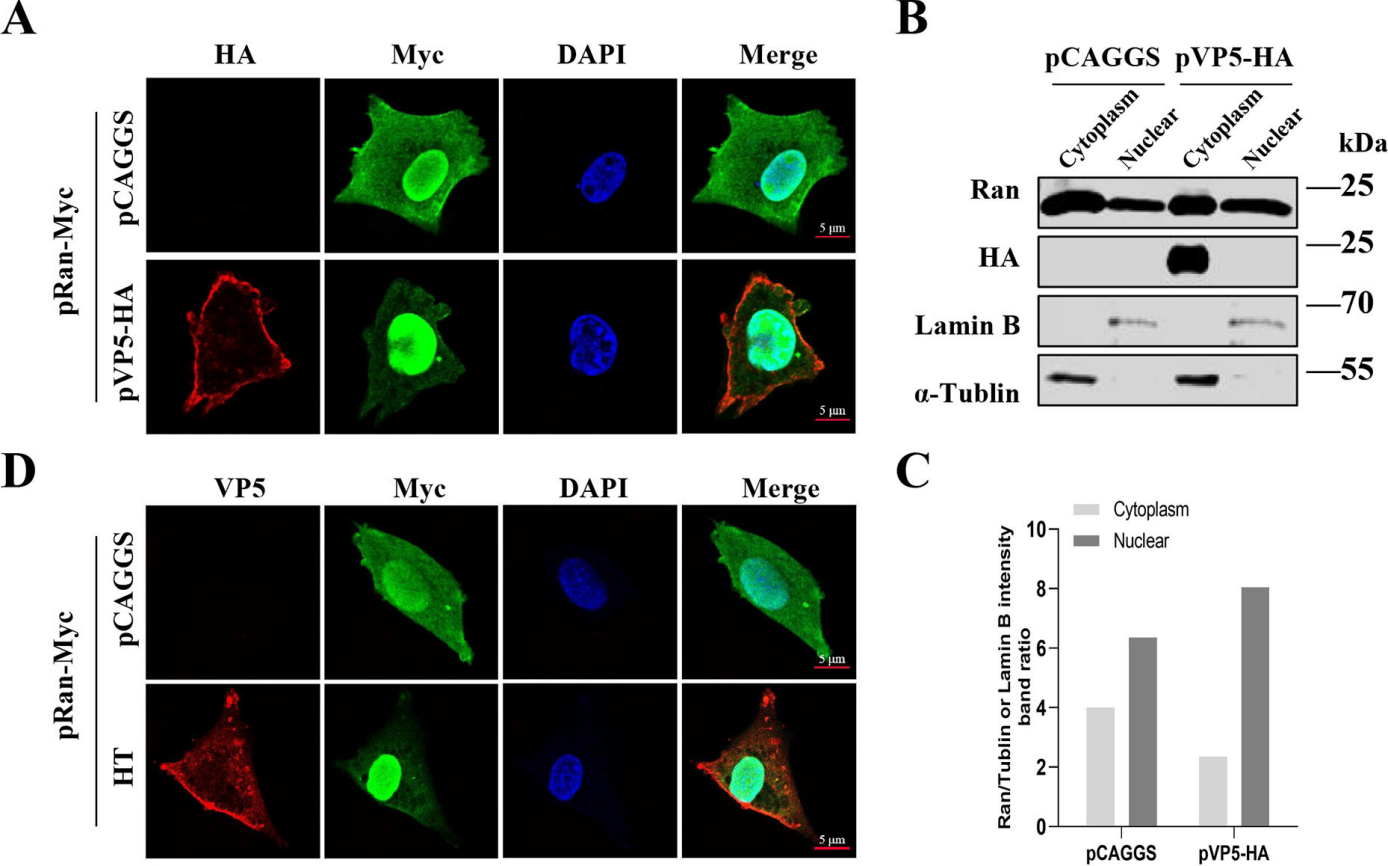
VP5 interferes with the nucleocytoplasmic localization of Ran proteins. (**A**) Representative images of DF1 cells co-transfected with recombinant plasmids expressing Ran (pRan-Myc) and either pVP5-HA or pCAGGS are presented. (**B**) DF1 cells were transfected with an empty vector or pVP5-HA for 24 h. Cell lysates were collected and separated into cytoplasmic and nuclear extracts, and Ran distribution in the cytoplasm and nucleus was determined by Western blotting. (**C**) Relative Ran intensity was quantified corresponding to Fig. 7B. (**D**) Representative images of DF1 cells transfected with recombinant plasmids expressing Ran (pRan-Myc) and infected with either IBDV or not are presented.

The active nucleocytoplasmic transport of proteins in eukaryotes relies on the Ran-GTPase system, which regulates cargo-receptor interactions. Proteins need to interact with an extended family of importin-related receptors to chaperone trafficking, and the small GTPase Ran controls the association of cargo with these receptors that bind to guanine nucleotides (GTP or GDP) (15, 32). To investigate how VP5 interferes with nucleocytoplasmic transport, we conducted co-immunoprecipitation (co-IP) experiments to examine whether VP5 directly interacts with nuclear transport receptors (KPNA1, KPNA2, KPNA3, KPNA4, KPNA5, KPNA6, KPNB1) and Ran. However, these interactions yielded negative results (data not shown). Another crucial factor is the cycling of the GTPase Ran, which switches between its GTP-bound and GDP-bound forms. This cycling maintains steep concentration gradients of different binding forms on the NE, enabling the regulation of cargo delivery and separation (9). DF1 cells were co-transfected with pRan-myc and either an empty vector or a recombinant plasmid expressing VP5 for 24 h. Then, the cells were fixed and immunolabeled to observe the intracellular distribution of Ran. The results demonstrated that VP5 leads to an aberrant redistribution of Ran between the cell nucleus and cytoplasm (Fig. 7A; Fig. S2A). To investigate the localization of endogenous Ran protein in the presence of VP5, corresponding Western blotting experiments were conducted to detect (Fig. 7B) and quantify (Fig. 7C) the levels of Ran in the cell nucleus and cytoplasm, which were consistent with the above results. It was noted that the phenomenon was also observed in another experiment involving viral infection. IBDV infection induced an anomalous distribution of Ran proteins between the nucleus and cytoplasm (Fig. 7D; Fig. S2B). Therefore, these findings suggest that IBDV VP5 may interfere with nucleocytoplasmic transport by specifically targeting the aberrant redistribution of Ran between the cell nucleus and cytoplasm.

**Fig 8:**
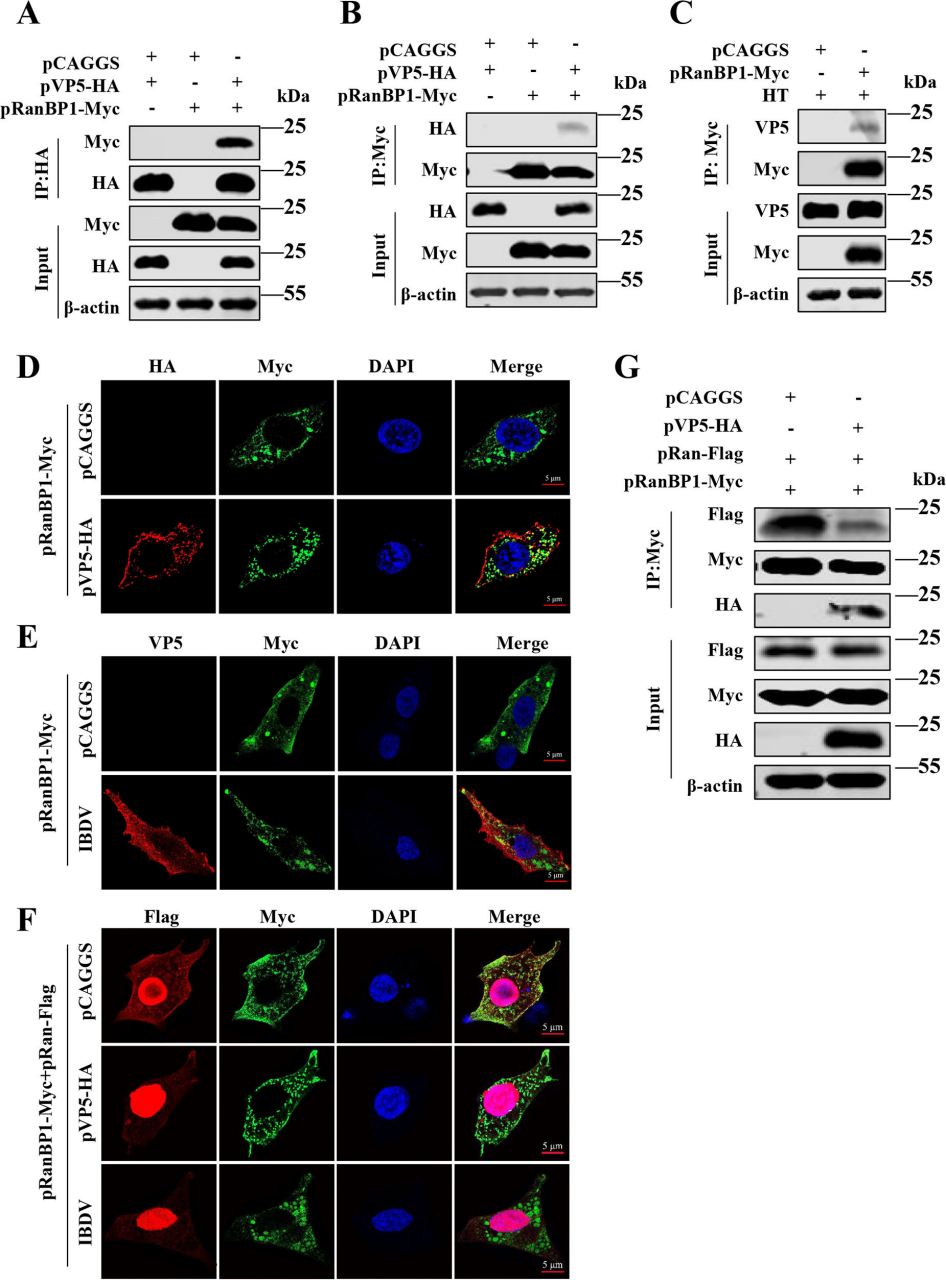
VP5 perturbs nucleocytoplasmic transport by disrupting the Ran cycle through interaction with RanBP1. (**A–C**) The co-IP assays were conducted to detect the interaction between RanBP1 and VP5. DF1 cells were transfected with pRanBP1-Myc (1.5 µg /1 × 10^6^ cells) and pVP5-HA (0.5 µg /1 × 10^6^ cells), or an empty vector (0.5 µg /1 × 10^6^ cells) for 24 h. The lysates were incubated with mouse anti-HA (**A**) or anti-Myc antibodies (**B**) at 4°C overnight. Protein G Sepharose beads were added and incubated for another 6 h. The beads were washed six times and boiled with SDS loading buffer for 10 min before analysis by Western blotting with the indicated antibodies. (**C**) DF1 cells were transfected with pRanBP1-Myc for 12 h and infected with IBDV for 24 h. The lysates were incubated with anti-Myc antibodies at 4°C overnight, and the same co-IP experimental steps were repeated as above. (**D and E**) Confocal assays were used to assess the colocalization between RanBP1 and VP5. DF1 cells were transfected with pRanBP1-Myc and pVP5-HA or an empty vector for 24 h (**D**) or transfected with pRanBP1-Myc and infected with IBDV (**E**) and then fixed and processed for dual labeling. RanBP1 (green) and VP5 (red) proteins were visualized by immunostaining with mouse anti-Myc and rabbit anti-HA antibodies. Cell nuclei were counterstained with DAPI (blue). The areas of colocalization in merged images are shown in yellow. (**F and G**) VP5 impacts the binding ability of Ran to RanBP1. The plasmid combinations pRanBP1-Myc/pRan-Flag/pVP5-HA or pRanBP1-Myc/pRan-Flag/pCAGGS were transfected into DF1 cells for 24 h, respectively. In another experimental group, DF1 cells were transfected with the plasmid combination pRanBP1-Myc/pRan-Flag for 4 h and then infected with IBDV for 20 h. Then, cells were fixed and processed for dual labeling (**F**). RanBP1 (green) and Ran (red) proteins were visualized by immunostaining with mouse anti-Myc and rabbit anti-Flag antibodies. Another set of samples under the same experimental conditions as Fig. 8F was quantitatively analyzed by co-IP assay (**G**) to determine the binding ability of Ran to RanBP1.

The guanine nucleotide exchange factor RCC1 promotes RanGDP/GTP exchange in the cell nucleus, while RanGAP and RanBP1, located in the cytoplasm, accelerate the slow intrinsic hydrolysis rate of RanGTP, cycling the complex back to the GDP form. These Ran-related factors, separated by the NE, maintain the cycling of Ran and the concentration gradients of the different binding forms. Any activity that disrupts GDP/GTP exchange has the potential to perturb the Ran cycling across the NE and the required cellular gradient, leading to the halt of active trafficking. To determine which specific step of the Ran pathway is impeded by VP5 during IBDV infection, we conducted further co-IP experiments and discovered a direct interaction between VP5 and the Ran-GTP-binding protein RanBP1 under conditions of VP5 protein expression (Fig. 8A and B) or IBDV infection (Fig. 8C). Correspondingly, the confocal experiments were performed at 24 h post-transfection, observing significant colocalization of the two proteins in the cytoplasm under conditions of VP5 protein expression (Fig. 8D; Fig. S3A) or IBDV infection (Fig. 8E; Fig. S3B). The data could be explained if VP5 either prevented RanGTP hydrolysis or accelerated RanGTP hydrolysis.

Nevertheless, both of these scenarios would disrupt the RanGDP/GTP gradient and interfere with host nucleocytoplasmic transport. RanBP1 stimulates the enzymatic activity of the Ran GTPase-activating protein RanGAP, allowing RanGAP-activated GTP hydrolysis on Ran. In subsequent experiments, we demonstrated that VP5 significantly disrupts the colocalization of RanBP1 and Ran proteins in the cytoplasm (Fig. 8F; Fig. S3C), which was further confirmed by co-IP experiments (Fig. 8G). Although, in the absence of precise measurements of GDP/GTP exchange in cells and cell-free extracts, it is challenging for us to ascertain this scenario, a more plausible explanation is that VP5 inhibits the hydrolysis of RanGTP by competitively binding to RanBP1, as predicted by the former possibility. In conclusion, VP5 transcription-dependent shutoff activity is achieved by disrupting the Ran cycle, thereby interfering with nucleocytoplasmic transport.

### VP5 with a shutoff function promotes IBDV infection *in vivo*

**Fig 9:**
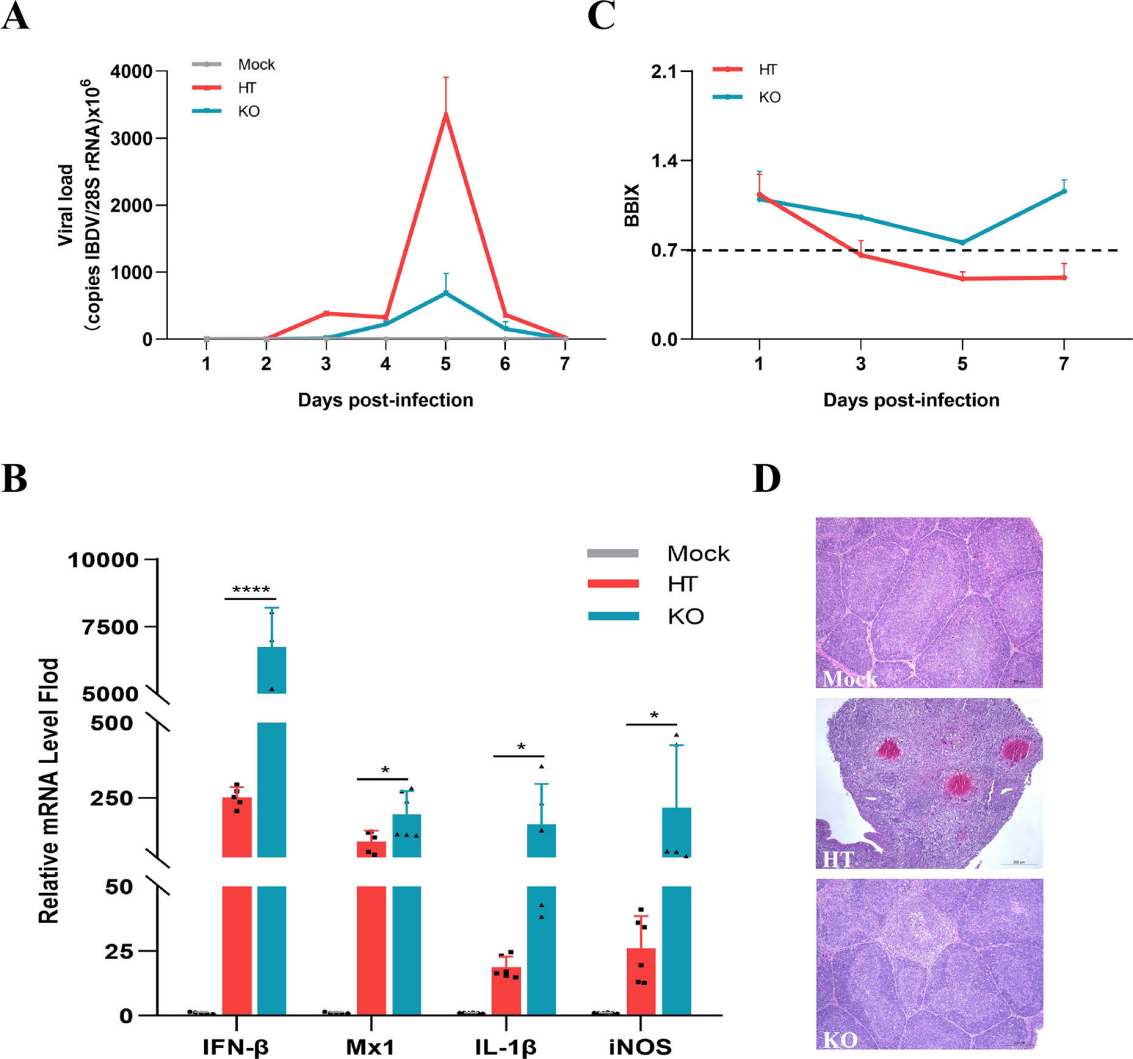
The pathogenicity comparison of IBDV with or without VP5 *in vivo.* (**A**) The growth kinetics of IBDV. The SPF chickens were infected with 10^6^ TCID_50_ of either the HT or KO strains, and a mock control group was treated with phosphate buffered saline (PBS). The viral copies in the bursa were quantified at 1-d intervals from d 1 to d 7 using RT-qPCR. (**B**) The expression levels of major cytokines (IFN-β, Mx1, iNOS, and IL-1β) in the bursa were analyzed at 5 dpi using RT-qPCR. (**C**) The bursa-weight:body-weight index (BBIX) was monitored at 1, 3, 5, and 7 dpi. (**D**) Histopathological examination of the bursa was performed to assess any pathological changes. *P < 0.05, ****P < 0.0001.

To investigate whether IBDV infection could induce host shutoff *in vivo*, we compared the biological characteristics of HT and KO strains using an infection model of specific-pathogen-free (SPF) chickens. The virus titers in the target organ, bursa, were measured at different time points. The HT strain showed a gradual increase in virus titer, reaching its highest value at 5 d post-infection (dpi), followed by a gradual decrease. Correspondingly, the KO strain without VP5 had a similar growth kinetics trend, but the titer was significantly lower, which is 7.2 times lower than the HT group at the peak point of 5 dpi (Fig. 9A). The cytokines in the bursa were detected. HT strain infection significantly suppressed the transcription of major cytokines, including IFN-β, Mx1, iNOS, and IL-1β, as shown in Fig. 9B. However, the KO strain, without VP5 shutoff activity, did not impede host cytokine synthesis, which might restore host immune response activity while resisting viral infection.

The pathogenicity of the two strains was also compared. The HT strain induced persistent atrophy of the bursa after 3 dpi, while the bursa-weight:body-weight index (BBIX) of the KO strain did not reach the definition of atrophy (Fig. 9C). Additionally, the histopathological analysis revealed that the HT strain caused comprehensive bursal atrophy, blurred borders, and multiple necroses of bursal follicles, whereas the KO strain-infected group only exhibited lymphopenia in individual follicles (Fig. 9D). These results suggest that the KO strain lacking shutoff activity lost the ability to restrict the expression of host antiviral cytokines, thereby attenuating viral replication and infection *in vivo*. Thus, IBDV can antagonize the host immune responses by suppressing host transcription through VP5 with a shutoff function, which represents a novel mechanism for IBDV to establish infection and evade host immunity.

## DISCUSSION

During viral infection, it is common for viruses to manipulate the expression profile of host genes to redirect cellular resources to support their replication while simultaneously suppressing the cellular antiviral response (33, 34). Similarly, in the case of IBDV, which is capable of overcoming host defenses and establishing infection within a short period, such a phenomenon may also be involved. In this study, we confirmed for the first time that IBDV infection is capable of globally suppressing host protein synthesis (Fig. 1). Further investigation into the underlying cause of IBDV-induced host shutoff revealed that viral VP5 plays a crucial role in this process. Additionally, the shutoff activity of VP5, a conserved viral protein, is prevalent in different representative IBDV strains. Our study unveils a novel strategy by which IBDV infection manipulates the expression of host genes through VP5 shutoff activity, which likely plays a critical role in the viral evasion of host immune responses and the efficient establishment of viral infection. To the best of our knowledge, this is the first report of *Birnaviridae* viruses inducing shutoff of host protein synthesis.

Viruses have evolved complex mechanisms to trigger host shutoff through interference with different stages of host gene expression (17, 33). In this study, we aimed to identify the pathway by which IBDV induces host shutoff. Obstructing host mRNA translation is a frequent strategy employed by viruses, with many translational arrest strategies targeting translation initiation through alterations in the eIF2-GTP-tRNAiMet ternary complex availability via phosphorylation of eIF2α or interference with the initial cap-binding step mediated by eIF4F. For example, murine norovirus (MNV) infection has been reported to progressively increase phosphorylated eIF2α, resulting in the suppression of host translation (1). Additionally, the nucleocapsid protein (NP) of Newcastle disease virus interacts with eIF4E to inhibit host protein translation (34). Furthermore, the Nsp1 protein of severe acute respiratory syndrome coronavirus 2 binds to the 40S ribosomal subunit, and its C-terminal domain physically obstructs the mRNA entry channel, leading to a reduction in translation (35–37). Therefore, as part of our investigation, we initially attempted to verify whether the shutoff activity of VP5 inhibits host translation initiation through an increase in phosphorylation of eIF2α. However, the results were negative, as shown in Fig. 3B. Subsequently, we sought to investigate the impact of VP5 on translation by directly evaluating its influence on mRNA. Our findings indicated that the shutoff activity of VP5 on host protein expression was present in the plasmid-transfection group but not in the mRNA-transfection group (Fig. 3), which negates the possibility of VP5 suppressing host protein synthesis by targeting the stage of translation.

Viruses employ several mechanisms to manipulate host gene expression at the mRNA level, including interference with host transcription and selective degradation of host mRNA. For instance, influenza A viruses (IAV) utilize the ribonuclease active protein PA-X to facilitate host RNA degradation, leading to the suppression of host protein synthesis (38). Additionally, the NS1 protein of IAV induces host shutoff by blocking the function of cleavage-polyadenylation specificity factor 30 (39, 40). Similarly, the herpes simplex virus (HSV) ICP27 protein regulates alternative pre-mRNA polyadenylation and splicing to decrease the mRNA level (41), while the vhs protein of HSV mediates widespread degradation of host mRNAs, inducing host shutoff (42). In our study, we discovered that blocking transcription with ActD could counteract the sustained reduction of host endogenous GAPDH mRNA and exogenous Rluc mRNA triggered by VP5 (Fig. 4C and D). Furthermore, our cellular nuclear-cytoplasmic fractionation experiments provided evidence of a significant reduction in the ratio of mRNA within the nucleus induced by the viral VP5. These results unequivocally establish that the host shutoff induced by IBDV VP5 operates through a transcription-dependent mechanism independent of the degradation of host mRNA.

During IBDV infection, VP5 does not enter the cell nucleus but targets Ran, resulting in its aberrant redistribution between the nucleus and cytoplasm. The active nucleocytoplasmic transport relies on the Ran-GTPase system, which regulates the interaction between cargo and receptors (9). Ran plays a critical role in directing cargo transport by creating a steep concentration gradient of GTP-bound and GDP-bound forms at the NE. Disrupting GDP/GTP exchange or the cycling of Ran can potentially disturb the cellular gradients necessary for efficient passage through the NE, consequently impeding active cellular transport. In our experiments, we observed VP5 interfering with the nuclear translocation of Cas9 protein, carrying nuclear localization signals. These findings underscore the broader impact of VP5 on nucleocytoplasmic transport activity in general.

Furthermore, our investigations confirmed that VP5 significantly disrupts the colocalization of Ran and RanBP1 in the cytoplasm by competitively binding to RanBP1. This interaction may disturb the cycling of Ran, leading to the obstruction of VP5-dependent nucleocytoplasmic transport and subsequent inhibition of global host protein synthesis. Notably, IBDV infection can trigger the formation of discrete viral factories in the cytoplasm for replication (43), independently of the host nucleocytoplasmic transport system. This finding may explain our earlier observation that IBDV infection can induce the shutoff of host protein synthesis, while viral proteins can bypass this restriction. Such a strategy could effectively restrict the host’s immune response and promote viral infections.

Viruses often utilize widespread suppression of host gene expression to evade the immune response (28, 29). For instance, the shutoff activity of IAV’s NS1 protein targets genes involved in IFN sensing and signaling, inflammatory response, and chemokine/cytokine-mediated signaling pathways, effectively countering innate immune responses (44). Similarly, MNV-induced host shutoff prevents infected cells from producing major cytokines, thereby facilitating viral infection (1). In the case of IBDV infection, acute damage to the bursa is considered a key mechanism for the virus to suppress host immune responses by impairing humoral and cellular immune defenses. We are particularly interested in whether IBDV can also antagonize the host’s innate immune response by suppressing the expression of cytokines in response to viral stimulation of host cells. As anticipated, VP5 induces host shutoff during viral infection, leading to a limited expression of crucial antiviral cytokines. Simultaneously, the parental strain of IBDV exhibits earlier attainment of the plateau phase and achieves higher viral titers compared to the VP5-deleted strain.

Interestingly, compared to the VP5-deleted strain, we observed a significant suppression of antiviral factors, such as IFN-β and the IFN-stimulated gene Mx1, in the infection group with the parental strain of IBDV. This finding suggests that IBDV can suppress the natural antiviral immune response through VP5-mediated host shutoff. Additionally, the expression of pro-inflammatory factors, IL-1β and iNOS, in the infection group with the parental strain was also notably restricted. Prior research indicates that uncontrolled acute inflammatory response caused by IBDV infection may trigger a “cytokine storm,” leading to severe consequences like sepsis and host death (2). In contrast, within normal limits, the inflammatory response promotes phagocytosis and clearance of IBDV. *In vivo,* although the KO strain infection triggered a higher level of inflammatory factors’ expression, it did not cause more severe inflammation and pathological damage; rather, it effectively restricted the viral titer in the bursa. These results suggest that the “cytokine storm” is a consequence of an etiologically complex immune imbalance, and the increased pro-inflammatory cytokines upregulated by the KO strain may have played a positive role in limiting IBDV infection. Thus, these findings demonstrate that VP5 decreases the host’s sensitivity to viral stimulation, suggesting a novel mechanism for IBDV-induced innate immune suppression. Furthermore, the study suggests that VP5 may play a crucial role in limiting excessive immune responses.

The disruption of nucleocytoplasmic transport by VP5 subsequently impairs the nuclear translocation of host proteins, including but not limited to the transcription factors. The suppression of p65 and IRF7 nuclear import by VP5, leading to the decrease of pro-inflammatory cytokines and IFNs, may not be specific but rather a general consequence of VP5-dependent disruption of the Ran gradient and subsequent inhibition of host protein synthesis. About the direct link between the disruption of the Ran protein cycle and the translocation of p65 and IRF7, given the current limitations of some conditions, such as the absence of precise measurements of GDP/GTP exchange in cells and cell-free extracts, more in-depth mechanisms need to be further explored in the future.

**Fig 10:**
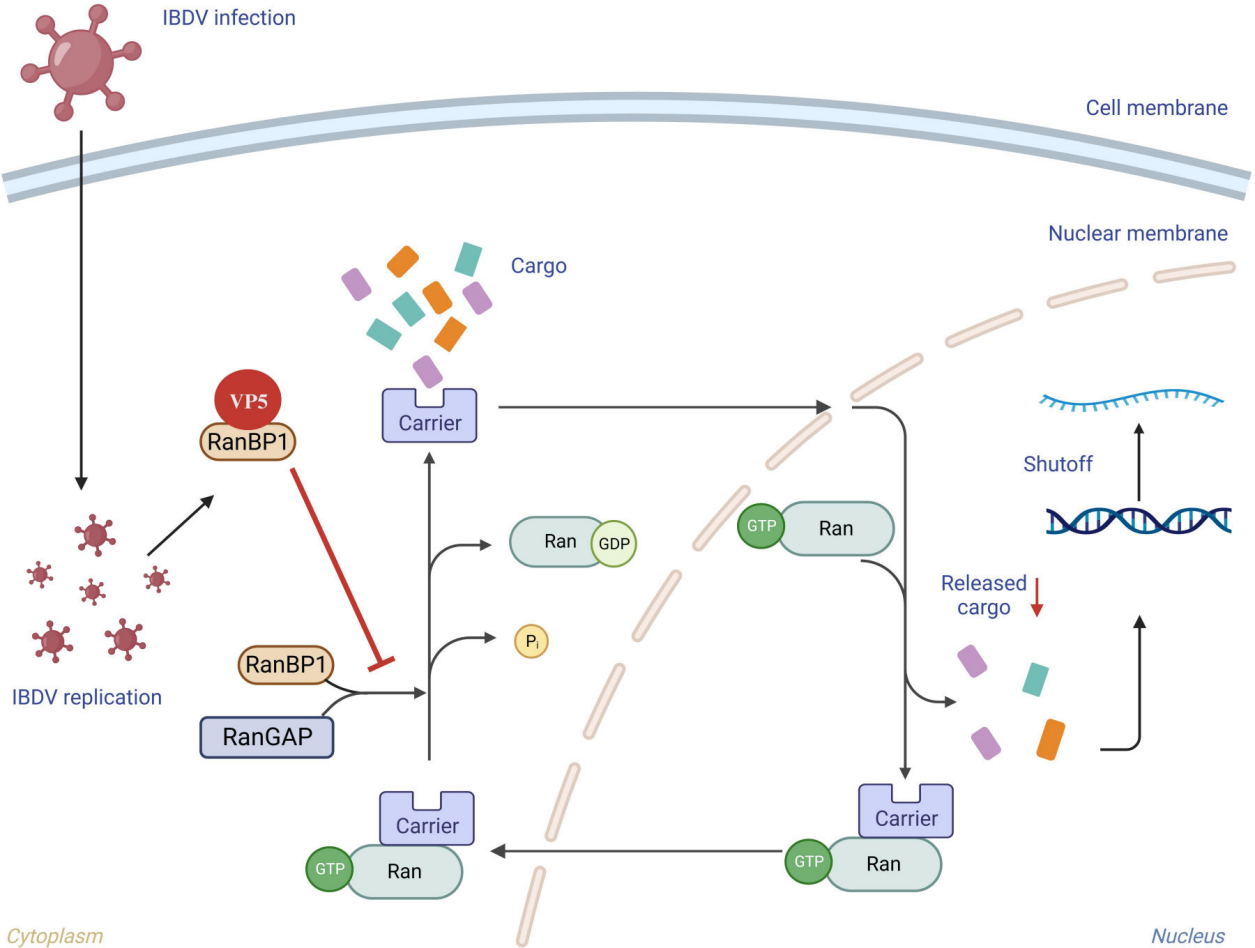
Schematic model of VP5-mediated host shutoff via transcriptional interference during IBDV infection. VP5 competitively binds with RanBP1, leading to disruption of the Ran protein cycle and consequently interfering with nucleocytoplasmic transport, which ultimately results in the suppression of host gene transcription.

In summary, we demonstrated that IBDV can induce a host protein synthesis shutoff. This novel phenomenon is mediated by the viral protein VP5, which effectively halts cytokine production and promotes viral infection. Notably, IBDV selectively targets and inhibits host gene transcription through VP5-induced shutoff, independent of cellular protein degradation pathways or protein translation. VP5 achieves this effect by competitively binding with RanBP1, thereby disrupting the Ran protein cycle and interfering with nucleocytoplasmic transport, resulting in the suppression of host gene transcription (Fig. 10). Interestingly, viral proteins may overcome this restriction by being synthesized in cytoplasmic viral factories. This newly unveiled strategy highlights how IBDV skillfully manipulates host gene expression to evade immune responses and establish successful viral infections.

## MATERIALS AND METHODS

### Cells and viruses

Chicken fibroblast cells DF1 were cultured in Dulbecco’s modified minimal essential medium (Gibco) supplemented with 10% fetal bovine serum (Gibco), 100 U/mL penicillin, and 100 mg/mL streptomycin. The cells were maintained in a humidified incubator at 38.5°C with 5% CO_2_. IBDV representative epidemic strains, including HLJ0504 (45), SHG19 (46), Gx, and Gt (47), were isolated and identified by the Avian Immunosuppressive Disease Research Group at Harbin Veterinary Research Institute, Chinese Academy of Agricultural Sciences, also known as “our laboratory” in this paper. The rescued HLJ0504 strain (referred to as HT) that can adapt to cells and its VP5 deletion strain (referred to as KO) with a mutated initiation codon of VP5 were rescued using reverse genetics (48) and preserved in our laboratory.

### Antibodies and reagents

Antibodies including mouse anti-Flag (catalog no. F1804), mouse anti-HA (catalog no. H9658), rabbit anti-HA (catalog no. H6908), mouse anti-c-Myc (catalog no. M4439), rabbit anti-c-Myc (catalog no. SAB4301136), mouse anti-puromycin (catalog no. MABE343), mouse anti-Renilla luciferase (catalog no. MAB4400), and mouse anti-actin (catalog no. A1978) were purchased from Sigma-Aldrich; rabbit anti-eIF2α (catalog no. 11170-1-AP) and rabbit anti-p-eIF2α (catalog no. 28740-1-AP) were from Proteintech; rabbit anti-Ran (catalog no. AB233762), rabbit anti-Lamin B1 (catalog no. AB229025), and rabbit anti-Tubulin (catalog no. EPR13796) were from Abcam. The secondary antibodies goat anti-rabbit IgG H&L (catalog no. A-11008) and goat anti-mouse IgG H&L (catalog no. A11003) were purchased from Invitrogen; IRDye 680RD goat anti-rabbit IgG H&L (catalog no. 926-68071) and IRDye 800CW goat anti-mouse antibody (catalog no. 926-32210) from LiCor Bio-Sciences. The mouse monoclonal antibodies against VP1 and VP5 of IBDV were produced and preserved in our laboratory. Actinomycin D (HY-17559), puromycin (HY-B1743S), the proteasome inhibitor MG132 (HY-13259), the autophagy inhibitor 3-MA (HY-19312), and the apoptosis inhibitor Z-VAD-FMK (HY-16658B) were purchased from MedChem Express. In addition, TransIT-X2 Dynamic Delivery System (MIR6000, Mirusbio), THUNDERBIRD SYBR qPCR Mix (QPS-201, TOYOBO), Premix Ex Taq (R390B, TaKaRa), HiScript II QRT SuperMix for qPCR (R223-01, Vazyme), NE-PER Nuclear and Cytoplasmic Extraction Reagents (78833, ThermoFisher Scientific), and PARIS Kit (AM1921, ThermoFisher Scientific) were used.

### Construction of plasmids

Plasmids were constructed for the viral genes VP1, VP2, VP3, VP4, and VP5 from IBDV strain HLJ0504 (GenBank no: GQ451330, GQ451331). These genes were amplified and cloned into the pCAGGS vector, each with an HA-tag fused to the N-terminal. The corresponding recombinant plasmids were designated as pVP1-HA, pVP2-HA, pVP3-HA, pVP4-HA, and pVP5-HA, respectively. Additionally, plasmids encoding chicken Ran (GenBank no. NM_205258), RanBP1 (GenBank no. NM_001396325), and IRF7 (GenBank no. NM_205372) were constructed by cloning the synthesized sequences into pCAGGS vectors with Myc or Flag tags. For Rluc expression, recombinant plasmids were constructed using the pCAGGS backbone, named pRluc. The pEGFP-C1 vector, encoding EGFP driven by the cytomegalovirus promoter, was purchased from Clontech (catalog no. HG-VYC0084). The pMJ920 vector (catalog no. 42234), an expression of codon-optimized Cas9 fused with GFP, was purchased from Addgene.

### RT-qPCR

Whole-cell RNA was extracted from transfected, infected, or uninfected mock cells at indicated time points using the RNAiso Plus kit (9109, TaKaRa, Japan). For nucleocytoplasmic separation assay, cytoplasmic and nuclear RNA were extracted using PARIS Kit (AM1921, ThermoFisher Scientific). The extracted RNA was reverse transcribed into cDNA using HiScript II QRT SuperMix for qPCR (R223-01, Vazyme, China). Each sample was triplicated for relative abundance analysis of individual mRNA transcripts from the viral genome or host factors, with 28 s mRNA serving as the normalizing reference. The RT-qPCR amplification reaction utilized the SYBR Green qPCR Kit (QPS-201, TOYOBO, Japan) with the following cycling conditions: initial denaturation at 95°C for 2 min, followed by 40 cycles of 95°C for 5 s, 60°C for 30 s, and a melt curve. The results were analyzed using the 2^-ΔΔC^ method.

To determine the viral loads of IBDV in infected cells, the Premix Ex Taq (Probe qPCR; R390B, TaKaRa, Japan) was employed with cycling conditions of 48°C for 30 min and 95°C for 20 s, followed by 40 cycles of 95°C for 3 s and 60°C for 30 s. Specific primers and TaqMan probes for 28 s and the IBDV genome were synthesized by Invitrogen. The primers used in this study are available upon request.

### Western blotting

Whole-cell lysates were obtained by lysing cells in NP-40 lysis buffer (Beyotime) containing a protease inhibitor cocktail (Roche). For the nucleocytoplasmic separation assay, cytoplasmic and nuclear proteins were extracted using NE-PER nuclear and cytoplasmic extraction reagents (ThermoFisher Scientific). Cellular lysate protein samples were boiled with 5× SDS loading buffer (Beyotime) for 10 min and then separated using sodium dodecyl sulfate 10% or 12.5% polyacrylamide gel electrophoresis. Subsequently, the proteins were transferred onto a nitrocellulose membrane. After blocking the membrane with 5% (wt/vol) skim milk for 2 h, it was incubated with the indicated primary antibodies for 1.5 h at room temperature. The membrane was then washed three times with phosphate buffered saline with Tween 20 (PBST) and exposed to the corresponding secondary antibody: IRDye1 680RD goat anti-rabbit IgG (H + L) or IRDye 800CW goat anti-mouse IgG (H + L) for 50 min. Finally, the membrane was washed four times in PBST and scanned using an Odyssey Infrared Imaging System (Li-Cor Biosciences) for further analysis.

### Ribopuromycylation assay

Puromycin is a compound that incorporates newly translated polypeptides, resulting in the termination of the translation of full-length proteins. To assess the impact of IBDV infection on host protein synthesis and the shutoff activity of VP5 protein, we employed the ribopuromycylation assay, which enables visualization of translational activity through the use of the anti-puromycin antibody. Cells were treated with puromycin (5 µg/mL) for 25–30 min prior to whole-cell lysate collection. Subsequently, the cell lysates underwent Western blotting analysis.

### Viral growth kinetics

The multi-step viral growth kinetics of IBDV’s HT and KO strains were analyzed in DF1 cells. The cells were infected with the viruses at an MOI of 0.01 and cultured for up to 72 h. Infected samples were collected at 12-h intervals, and then tested for released virus titers of TCID_50_ using the Reed-Muench method. The virus growth kinetics were plotted based on the virus titers at different infection time points.

### Animal experiments

Sixty-three 3-week-old SPF chickens were randomly divided into three groups and infected with either the HT or KO strain of IBDV at 10^6^ TCID_50_/200 µL or 200 µL PBS, respectively. The viral loads in the collected bursae were measured at 1-d intervals from d 1 to d 7 using RT-qPCR. The chickens’ symptoms were recorded, and the BBIX was monitored to compare the virulence of the two strains. Bursa with a BBIX below 0.7 was considered atrophied, as described previously (49). Additionally, bursa samples from different groups were fixed in 10% neutral buffered formalin and stained with hematoxylin and eosin for further histopathological examination.

### Statistical analysis

Unless otherwise indicated, all bar graphs represent the arithmetic mean of three independent experiments (*n* = 3), with error bars denoting standard deviations. Statistical significance was determined using GraphPad Prism 8.0.1 (GraphPad Software, USA) with one-way analysis of variance. A *P*-value of less than 0.05 (*P* < 0.05) was considered statistically significant for all tests.

## SUPPLEMENTAL MATERIAL

Supplemental Figures mbio.03433-23-s0001.docx Fig. S1 to S3.

ASM does not own the copyrights to Supplemental Material that may be linked to, or accessed through, an article. The authors have granted ASM a non-exclusive, world-wide license to publish the Supplemental Material files. Please contact the corresponding author directly for reuse.
