# Supplementary material for: Infectious bursal disease virus VP5 triggers host shutoff in a transcription-dependent manner
Source: mBio. 2024 Jan 30;15(3):e03433-23. doi: 10.1128/mbio.03433-23 (PMC10936426; doi:10.1128/mbio.03433-23)
Supplement: Supplemental Figures — Fig. S1 to S3. [file mbio.03433-23-s0001.docx]

**
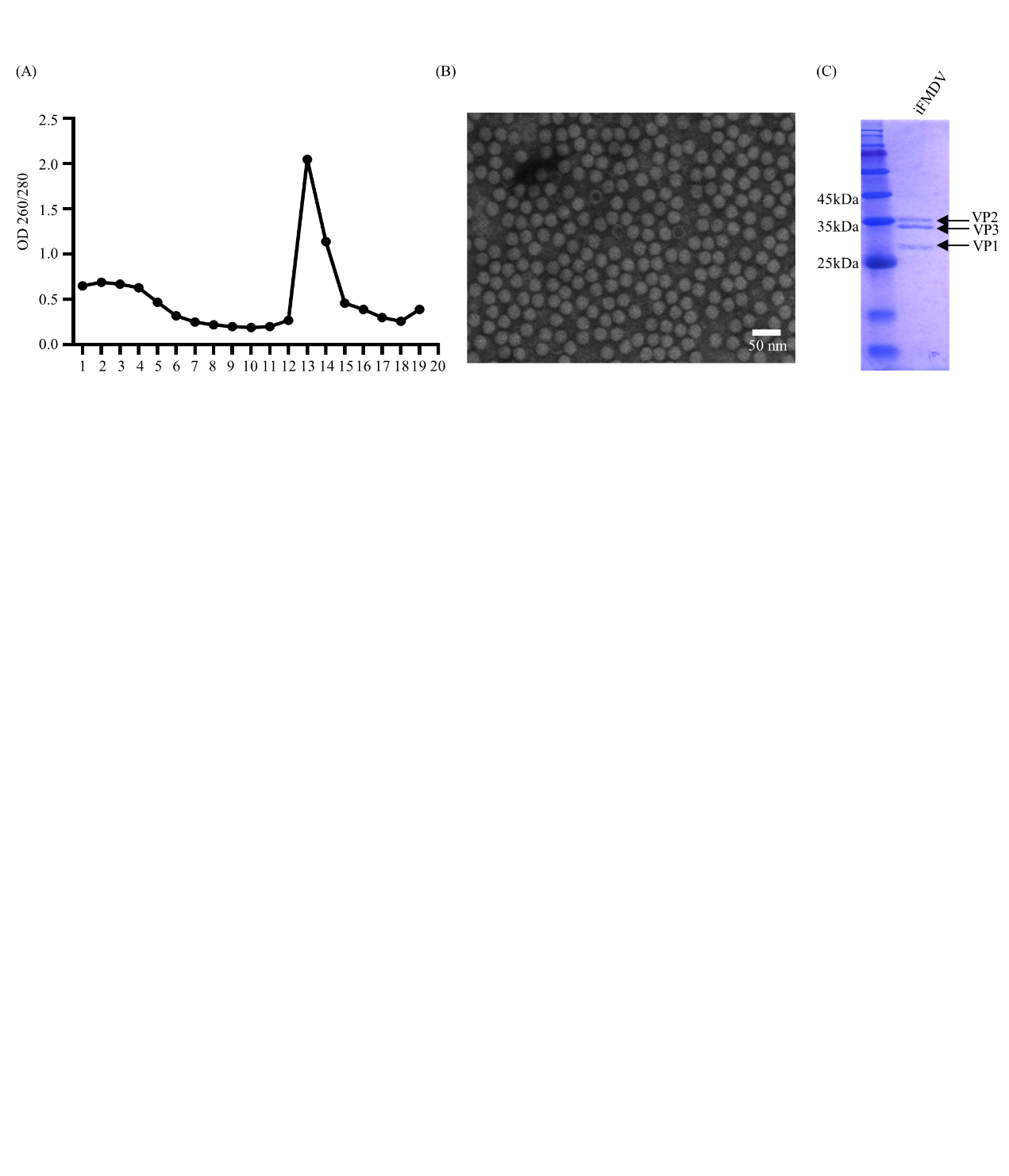
**

**Supplementary Fig. 1. Purification and characterization of FMDV capsids.**

(A and B) A negatively stained electron micrograph of the *OD_260/280_* peak values(A) collected from a 10 to 50% sucrose density gradient demonstrates the presence of full capsids(B), bar=50 nm. (C) VP1 (27 kDa), VP2 (36 kDa), and VP3 (31 kDa) bands were observed in the whole protein analyzed by SDS-PAGE. Lane M, molecular mass marker.


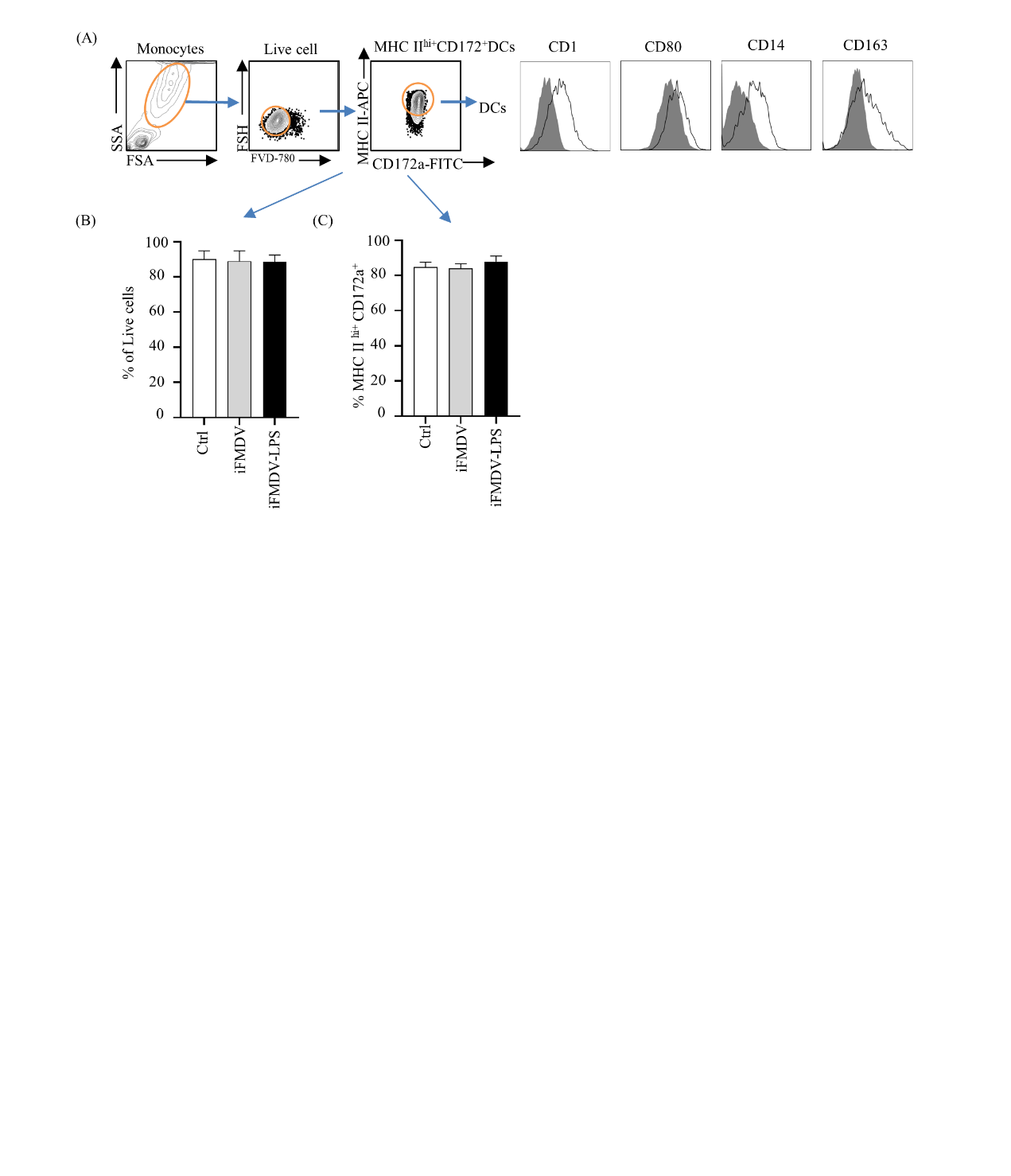


**Supplementary Fig. 2. Gating strategy of autologous monocyte-derived DCs.**

(A) Gating strategy for porcine MoDCs. Monocyte (blue) was gated according to FSC-A/SSC-A properties, and dead cells were excluded (red). DC cells were identified by co-expression of MHC II^hi^ and CD172a (orange) and further sub-gated. Representative histograms show CD1, CD80, CD14, and CD163 expression on MoDCs. Gray solid areas indicate fluorescence minus one control (FMO). (B and C) the percentage of live cells and DCs treated with iFMDV+LPS, iFMDV, and medium.


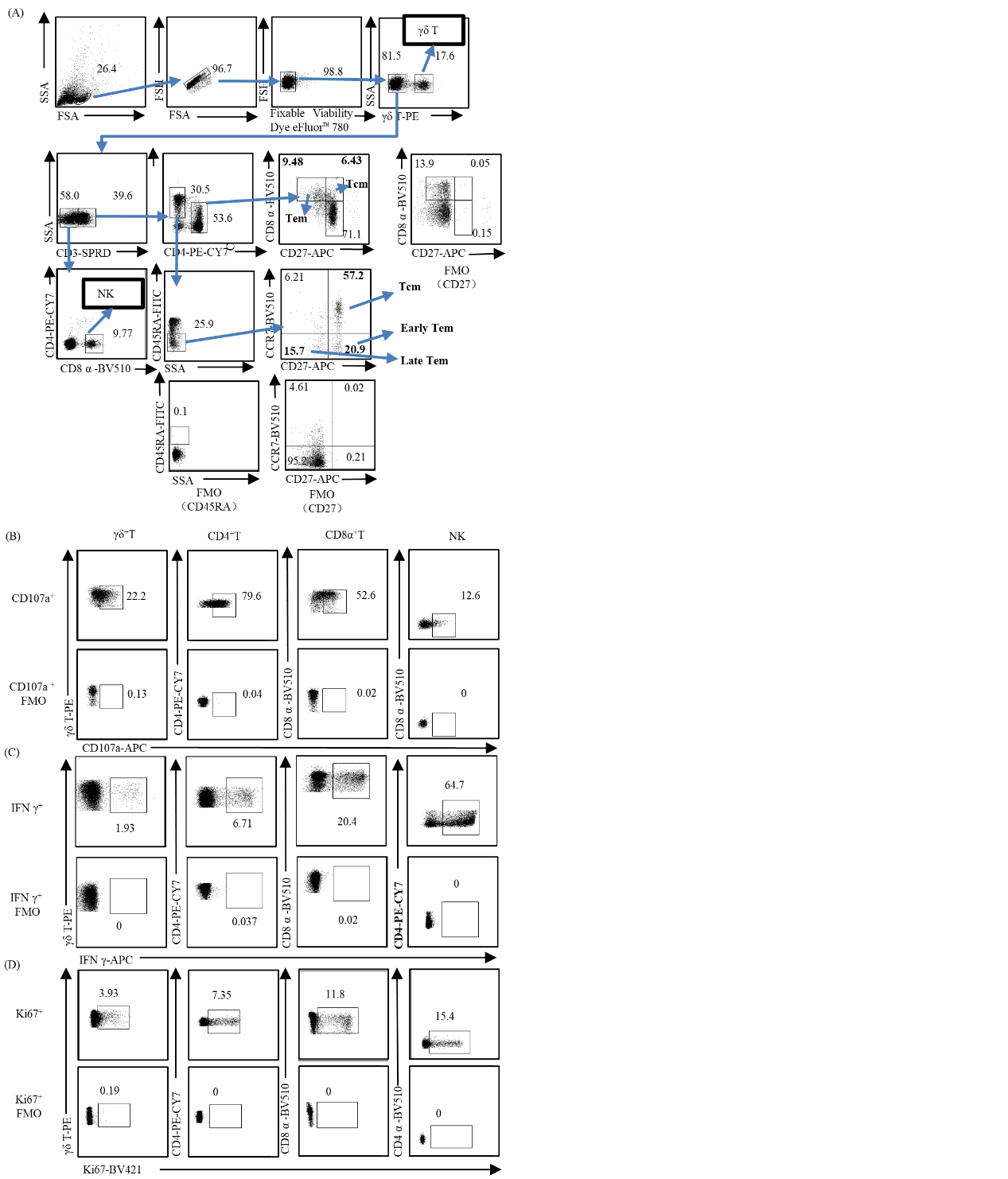


**Supplementary Fig. 3. Gating strategy of memory T cell, Intracellular cytokine, CD107a and Ki67 expression in flow cytometric analysis.**

(A) Gating strategy for distinct T cell subsets and NK cells. Lymphocytes were gated according to FSC-A/SSC-A properties; doublets and dead cells were excluded (Fixable Viability Dye eFluor™ 780). γδ T cells were identified on lymphocytes and excluded. NK cells were identified by γδ^-^CD3^-^CD4^-^CD8α^+^. CD4^+^ and CD8^+^ T cells were identified as γδ^-^CD3^+^CD4^+^ and γδ^-^CD3^+^CD4^-^CD8α^+^, respectively. Furthermore, CD4 T_CM_ (CD4^+^CD27^+^CD8α^+^), T_EM_ (CD4^+^CD27^-^CD8α^+^) and CD8 T_CM_ (CD45RA^-^CCR7^+^CD27^+^), early T_EM_ (CD45RA^-^CCR7^-^CD27^+^) and late T_EM_ (CD45RA^-^CD27^-^CCR7^+^) were identified on CD4^+^ and CD8^+^ T cell. FMO was set for CD27 and CD45RA. (B) Left to Right are representative dot-plots of CD107a-positive γδ T, CD4^+^ T, CD8α^+^ T, and NK cells and their FMO (lower). (C) Left to Right are representative dot-plots of IFN-γ-expressing γδ T, CD4^+^ T, CD8α^+^ T, and NK cells upon mitogen stimulation and their FMO. (D) Representative dot-plots of ki67-positive γδ T, CD4^+^ T, CD8α^+^ T, and NK cell and their FMO.


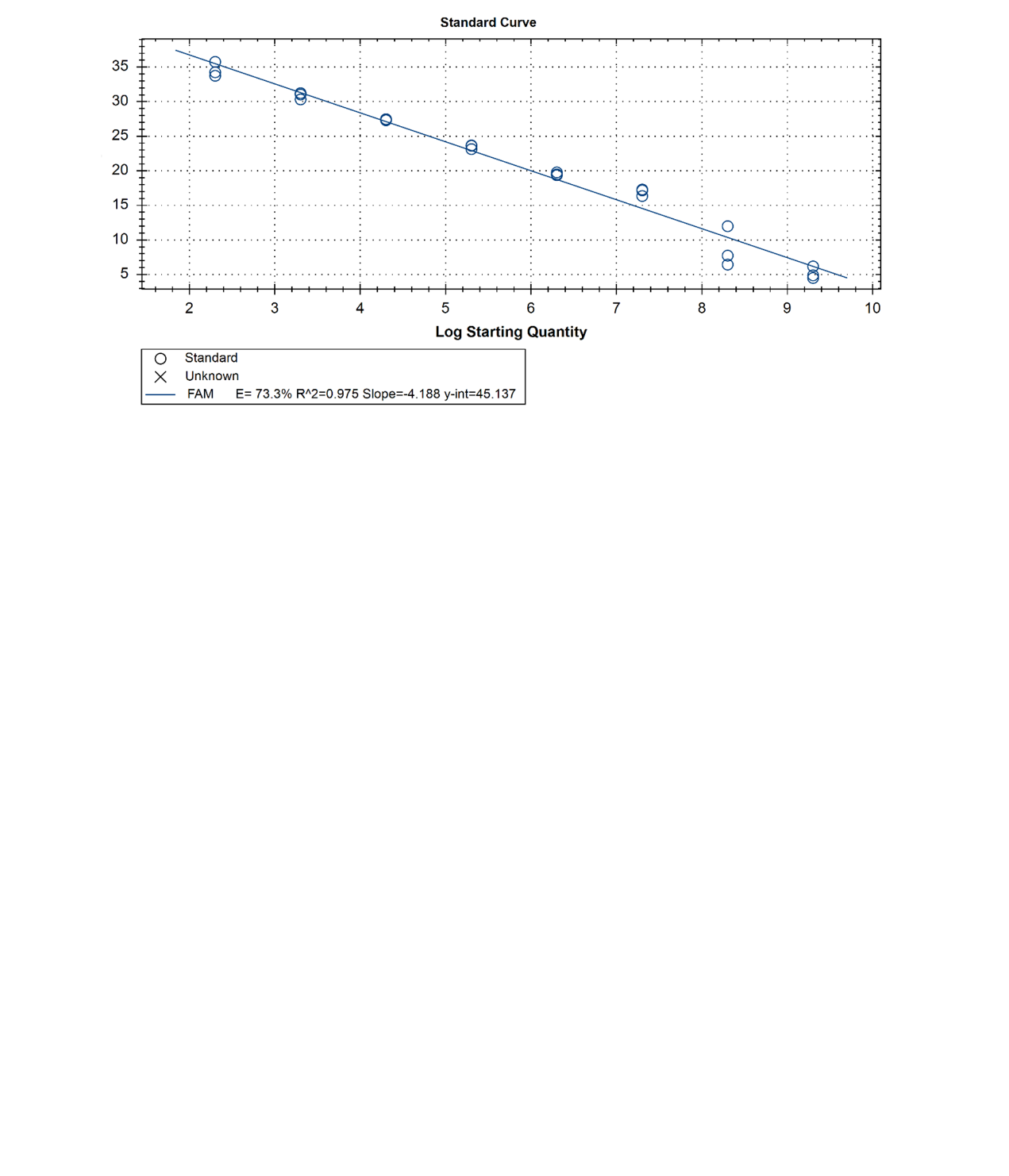


**Supplementary Fig. 4. Standard curve for FMDV RNA series dilution measured by qRT-PCR.**

FMDV RNA was extracted from previously titrated viral stocks, serially diluted (10-fold), and analyzed by TaqMan one-step quantitative reverse transcriptase PCR (qRT-PCR) to generate a standard curve.

| **Antigen** | **Clone** | **Isotype** | **Fluorochrome** | **Source of primary Ab** | **Details of secondary Ab** |
| --- | --- | --- | --- | --- | --- |
| **DC vaccine phenotype assay** | | | | | |
| CD172a | BL1H7 | Mouse IgG1 | FITC | Bio-rad |  |
| CD163 | 2A10/11 | Mouse IgG1 | PE | Bio-rad |  |
| CD80 | 16-10A1 | Armenian hamster IgG2 | Perc-cy5.5 | Biolegend |  |
| SLA II DQ | K274.3G8 | Mouse IgG1 | Purified | Bio-rad | Lightning-Linked APC,Abcam |
| SLA II DR | 2E9/13 | Mouse IgG2b | Purified | Bio-rad |  |
| CD14 | Mil2 | Mouse IgG2b | Purified | Bio-rad | ReadiLink™ Rapid mFluor™ Violet 450, AAT Bioquest |
| CD1 | 76-7-4 | Mouse IgG2a | Purified | Bio-rad | Lightning-Linked PE-CY7, Abcam |
| **Memory T cell assay** | | | | | |
| CD3 | BB23-8E6-8C8 | Mouse IgG2a | PerCP-cy5.5 | BD Pharmingen |  |
| CD4 | 74-12-4 | Mouse IgG2b | PE-Cy™7 | BD Pharmingen |  |
| CD8α | 76-2-11 | Mouse IgG2aκ | Biotin | Southernbiotech | BV 421™ |
| TCRγδ | MAC320 | Rat IgG2a | PE | BD Pharmingen |  |
| CCR7 | 3D12 | Rat IgG2a | BV510 | BD Pharmingen |  |
| CD27 | B30C7 | Mouse IgG1 | APC | Bio-rad |  |
| CD45RA | MIL13 | Mouse IgG1 | FITC | Bio-rad |  |
| **Live/dead cell and Streptavidin antibody** | | | | | |
| Streptavidin |  |  | BV 510™ | Biolegend |  |
| Streptavidin |  |  | BV 421™ | Biolegend |  |
| Fixable Viability Dye eFluor™ 780 |  |  | eFluor™ 780 | eBioscience™ |  |
| **CD107a assay** | | |  |  |  |
| CD107a | 4E9/11 | Mouse IgG1 | APC | Bio-rad |  |
| CD3 | BB23-8E6-8C8 | Mouse IgG2a | PerCP-cy5.5 | BD Pharmingen |  |
| CD4 | 74-12-4 | Mouse IgG2b | PE-Cy™7 | BD Pharmingen |  |
| CD8α | 76-2-11 | Mouse IgG2aκ | Biotin | Southernbiotech | BV 510™ |
| TCRγδ | MAC320 | Rat IgG2a | PE | BD Pharmingen |  |
| **ICS assay** | | |  |  |  |
| CD3 | BB23-8E6-8C8 | Mouse IgG2a | PerCP-cy5.5 | BD Pharmingen |  |
| CD4 | 74-12-4 | Mouse IgG2b | PE-Cy™7 | BD Pharmingen |  |
| CD8α | 76-2-11 | Mouse IgG2aκ | Biotin | Southernbiotech | BV 510™ |
| TCRγδ | MAC320 | Rat IgG2a | PE | BD Pharmingen |  |
| IFN-γ | P2G10 | Mouse IgG1 | APC | BD Pharmingen |  |
| **Ki67 proliferation assay** | | | | | |
| CD3 | BB23-8E6-8C8 | Mouse IgG2a | PerCP-cy5.5 | BD Pharmingen |  |
| CD4 | 74-12-4 | Mouse IgG2b | PE-Cy™7 | BD Pharmingen |  |
| CD8α | 76-2-11 | Mouse IgG2aκ | Biotin | Southernbiotech | BV 510™ |
| TCRγδ | MAC320 | Rat IgG2a | PE | BD Pharmingen |  |
| Ki67 | B56 | Mouse IgG1 | BV 421™ | BD Pharmingen |  |

**Supplementary Table 1. Antibodies used in ﬂow cytometric analyses of this study.**

| Clinical symptoms | Clinical score | Total score |
| --- | --- | --- |
| Left forefoot blisters | 1 | 5 |
| Right forefoot blisters | 1 |  |
| Left hindfoot blisters | 1 |  |
| Right hindfoot blisters | 1 |  |
| Mouth and nose blisters | 1 |  |

**Supplementary Table 2. Clinical Symptom Scoring.**
